# Functional impact of long Covid on work performance and return to work: a cross-sectional study

**DOI:** 10.11606/s1518-8787.2026060007097

**Published:** 2026-08-07

**Authors:** Fabio Cavalcanti Freitas, Vivian Cintra Sousa, Daniella de Melo, Diêgo Araújo de Moraes Ramos, Gabriela Sayuri Ochiai, Daniel Kolisch, Julia Costa Guimarães, Larissa Gomes da Silva, Erika Christina Gouveia e Silva, José Eduardo Pompeu, Ana Carolina Basso Schmitt

**Affiliations:** I Universidade de São Paulo Faculdade de Medicina Departamento de Fisioterapia, Fonoaudiologia e Terapia Ocupacional São Paulo SP Brasil Universidade de São Paulo. Faculdade de Medicina. Departamento de Fisioterapia, Fonoaudiologia e Terapia Ocupacional. São Paulo, SP, Brasil

**Keywords:** Post-Acute COVID-19 Syndrome, Functional Status, Work Performance, Return to Work

## Abstract

**OBJECTIVE:**

To evaluate the association between long Covid and socioeconomic, clinical, healthcare, and functional factors with reduced work performance and difficulty returning to work.

**METHODS:**

A cross-sectional study of individuals in São Paulo aged ≥ 18 years with long Covid (77.8%), with data collected between April 2023 and June 2024. A systematic random sample of severe and mild Covid-19 cases from March 2020 to January 2022. The severity of Covid-19 was classified according to the U.S. Centers for Disease Control and Prevention (CDC) guidelines. Prevalences were assessed, and a Poisson test was performed to examine the association between reduced work performance and difficulty returning to work and sociodemographic, clinical, healthcare-related, and functional factors considered significant (p ≤ 0.05). Among the participants, 43.9% experienced reduced work performance, and 22.2% had difficulty returning to work. Long Covid is defined as having two or more symptoms that develop and persist for more than four weeks after Covid-19, cannot be explained by an alternative diagnosis, and are assessed using a Likert scale for the intensity of self-reported symptoms (0–10). Participants’ functional status was assessed using scales for basic activities (Katz), instrumental activities (Lawton), functional limitations (WHO-DAS), and sarcopenia (SARC-F).

**RESULTS:**

Poorer work performance was associated with advanced age (PR = 2.20; 95%CI 1.27–3.80), as well as being the primary provider (PR = 1.55; 95%CI 1.05–2.29), and having moderate (OR = 2.99; 95%CI 1.60–5.58) or severe (OR = 4.88; 95%CI 2.59–9.19) functional impairment. For difficulty returning to work, the main association observed was severe functional impairment (OR = 4.33; 95%CI 1.58–11.80).

**CONCLUSION:**

It is noteworthy that advanced age and, particularly, severe functional impairment were significantly associated with poorer performance and difficulty returning to work in the studied population with long Covid.

## INTRODUCTION

SARS-CoV-2 is a coronavirus that causes Covid-19 infection and can present asymptomatically or with mild symptoms in approximately 80% of cases. However, it can cause an acute respiratory syndrome capable of progressing to severe respiratory failure in 5% to 10% of cases^1^. In general, factors associated with both the acute illness and lockdown measures are linked to muscle weakness, which, to some extent, reduces functional capacity to varying degrees, impacting quality of life, survival^2^, and work^3^.

Most people with Covid-19 notice an improvement in their symptoms a few days or weeks after the onset of the infection. However, in 10% to 20% of cases, “long Covid” develops, defined as two or more symptoms persisting for more than four weeks after the onset of infection and not attributable to an alternative diagnosis^4^. According to recent reviews, this delayed recovery from symptoms can last for more than seven months^2,4,5^.

People who have had Covid-19 and required hospitalization, particularly those admitted to intensive care units (ICUs), experience functional difficulties after discharge, including severe and prolonged muscle weakness, fatigue, joint stiffness, myopathy, critical illness neuropathy, dysphagia, neuropsychological problems, and impaired functionality, including worsened gait and mobility—conditions that hinder an early return to work^2^.

Even those workers who managed to return to work, despite having long Covid, face consequences such as reduced overall well-being, difficulty performing tasks they previously carried out, loss of concentration, and decreased work performance^6^.

The literature indicates that factors such as being female, advanced age, the presence of chronic diseases, dyspnea, and fatigue can impact work and hinder an immediate return to work following the development of severe Covid-19^7^. However, there are few studies linking the functional impacts of long Covid to work performance and the return to work^3^.

Consequently, it is necessary to estimate the prevalence of reduced work performance and difficulty returning to work, as well as to associate these with long Covid, socioeconomic, clinical, healthcare-related, and functional factors in people who have had Covid-19.

## METHODS

### Study Type and Population

A cross-sectional study was conducted in São Paulo, the city with the highest absolute number of Covid-19 cases in Brazil during the pandemic period^11^, using data on individuals diagnosed with Covid-19 recorded by the Secretaria Municipal de Saúde (Municipal Health Department) between March 2020 and February 2022, pursuant to approval under Opinion No. 5,053,884.

A total of 337,420 cases diagnosed with Covid-19, both severe and mild, were reported in the municipality of São Paulo. Severe cases were identified using the “Ficha de Registro Individual do Ministério da Saúde – Casos de Síndrome Respiratória Aguda Grave Hospitalizado (Ministry of Health Individual Registration Form—Hospitalized Cases of Severe Acute Respiratory Syndrome)”^12^; and mild cases using the “Ficha de Investigação de SG Suspeito de Doença pelo Coronavírus 2019 (Covid-19) (Investigation Form for Suspected Cases of Coronavirus Disease 2019 [Covid-19])”^13^.

The study included individuals aged 18 years or older, of both sexes, with a diagnosis of Covid-19 confirmed by a positive PCR test or serological test showing reactive IgM (Immunoglobulin M) and non-reactive IgG (Immunoglobulin G), who electronically signed the Informed Consent Form. Individuals with inconsistent information that made contact impossible were excluded, as were those who did not respond to the invitation to participate in the study.

The severity criteria adopted by the city of São Paulo were those outlined in the U.S. Centers for Disease Control and Prevention (CDC) guidelines^14^ and those of the Brazilian Ministry of Health. Cases were considered mild regardless of whether they presented with flu-like symptoms such as a runny nose, cough, sore throat, or headache. For severe cases of Covid-19, patients who exhibited one or more of the following signs were included: respiratory distress (respiratory rate > 30 breaths per minute), peripheral oxygen saturation < 93% on room air, diffuse abdominal pain, and hemodynamic instability.

Long Covid arising from mild and severe cases was classified according to the Ministry of Health^4^ and was identified when two or more symptoms persisted for more than four weeks after the onset of infection and could not be explained by an alternative diagnosis^4^.

### Sample

From a population of 337,420 reported cases (divided into severe and mild cases), a random selection of 25,026 individuals was made, with proportional representation of the population across the municipality’s six health regions, to be invited to participate. Of these, 775 people, 395 diagnosed with mild Covid-19 and 380 with severe Covid-19, participated in the study by completing the electronic questionnaires.

Data collection took place between April 2023 and June 2024, an average of 2.5 years after infection. Participants were invited to take part in the study via telephone. Those who agreed to participate electronically signed the Informed Consent Form and completed a self-administered questionnaire via a link sent by the researchers, which contained the information needed to fill out the form, as well as instructions for contacting the researchers by phone in case of questions or difficulties in completing it. Data security and storage were handled in accordance with the Lei Geral de Proteção de Dados Pessoais (LGPD – General Personal Data Protection Law).

To calculate the sample size, an expected prevalence of 30% of post-Covid-19 functional disability^15^ was considered, with a maximum acceptable error of 4% and a 95% confidence level. Based on these parameters, it was estimated that at least 504 people needed to be included in the study. The final sample, consisting of 775 individuals with mild and severe Covid-19 who completed the questionnaires, resulted in a statistical power of 99.0% to detect the expected prevalence.

### Variables

Data collection was conducted using a self-administered questionnaire, made available electronically via the RedCap® platform, and sent to participants who accepted the invitation. The questionnaire was designed to collect different categories of information relevant to the study:

- *Sociodemographic data*: These included age, sex, race/ethnicity, marital status, education level, employment status, household income, and status as the family’s primary caregiver.
- *Clinical data*: These included pre-existing comorbidities prior to Covid-19, the severity of acute Covid-19 infection (mild or severe), the time from the onset of the first symptoms to the date of the interview, and the presence of long Covid, defined as the persistence of two or more symptoms for more than four weeks after the onset of infection, not explained by another diagnosis.
- *Post-Covid symptoms*^17^: These included headache, muscle pain, joint pain, loss of balance, dizziness/vertigo, lack of concentration, physical malaise, fatigue, dyspnea, cough, and memory loss. The intensity of each self-reported symptom was quantified on a Likert scale^18^ ranging from 0 to 10, where 0 represented very mild symptoms and 10 represented very severe symptoms.
- *Clinical data*: Information was collected on hospital admission, ICU admission, and the need for invasive ventilatory support during the acute phase of Covid-19.
- *Functional assessment*: The questionnaire included standardized scales to assess participants’ functional capacity at the time of response:
- *Katz Index*^19^: used to assess independence in basic activities of daily living (BADL), such as bathing, dressing, using the toilet, continence, and eating.
- *Lawton Scale*^20^: applied to measure independence in instrumental activities of daily living (IADLs), which include more complex tasks such as using the telephone, shopping, preparing meals, housekeeping, doing laundry, using public transportation, managing medications, and handling money.
- WHODAS 2.0 (*World Health Organization Disability Assessment Schedule* 2.0) short form^21^: a comprehensive questionnaire on functionality that assesses the impact of a health condition on daily activities across different domains (cognition and communication, mobility, self-care, social relationships, activities of daily living, and participation).
- SARC-F^22^: a screening tool to assess increased risk of sarcopenia, based on five items (strength, assistance with walking, climbing stairs, rising from a chair, and falls).

### Assessment of Work Performance and Return to Work

The primary outcomes, “reduced work performance” and “difficulty returning to work”, were assessed using self-reported dichotomous (“yes” or “no”) questions included in the questionnaire and directed only at participants who reported having paid work prior to Covid-19 and who were neither retired nor unemployed at the time of the survey.

*Reduced work performance:* The question asked was: “Did your work performance decline after contracting Covid-19 due to your physical condition and/or your mental health condition?” A “yes” response indicated reduced performance.

*Difficulty returning to work:* The question was: “Did the physical and/or mental health sequelae resulting from your Covid-19 infection prevent you from returning to work?” A “yes” response indicated difficulty.

*Association between functional assessment scales and work-related outcomes:* Functional assessment scales (Katz, Lawton, WHODAS 2.0, SARC-F) were used to provide a more structured and standardized measure of the individuals’ functional status. Although the work-related outcomes were based on self-reports, the inclusion of these scales made it possible to:

- Corroborate the functional impact by verifying whether the difficulties reported at work were associated with objective impairments in the ability to perform basic and instrumental activities of daily living, as well as in overall functional well-being;
- Identify contributing factors, analyzing which specific aspects of functionality (e.g., mobility, self-care, and risk of sarcopenia) were most strongly associated with the perception of reduced performance or difficulty returning to work;
- Contextualize the findings, offering a more comprehensive view of the functional profile of affected workers, going beyond individual perception and incorporating a more clinical dimension into the assessment of the impact of long Covid in the workplace.

### Statistical Analysis

The data were analyzed using Stata 14. Categorical variables were presented as absolute and relative frequencies (%), while continuous variables were described using mean and standard deviation. The normality of the distribution was assessed using the Shapiro-Wilk test. Percentages and confidence intervals for reduced work performance and difficulty returning to work were calculated according to disease severity and the presence of long Covid.

A two-way analysis of variance (two-way ANOVA) was performed to assess the association between the intensity of persistent post-Covid-19 symptoms (on a scale of 0 to 10) and work performance (whether or not it influenced reduced performance), as well as disease severity (mild or severe), including the interaction term between these factors.

Likewise, to assess the association between the intensity of persistent post-Covid-19 symptoms (on a scale of 0 to 10) and difficulty returning to work (yes/no), with disease severity (mild or severe), including the interaction term between the factors.

The assumptions of normality of residuals and homogeneity of variances were considered. P-values < 0.05 were considered statistically significant.

The dependent variables were “reduced work performance”^16^ and “difficulty returning to work”^3^. Both were assessed using a dichotomous question (yes or no). For “reduced work performance,” participants were asked whether their work performance had decreased after Covid-19 infection due to physical and/or mental health conditions. For “difficulty returning to work,” the study investigated whether the physical and/or mental sequelae of Covid-19 prevented a return to work.

The independent variables considered included sociodemographic factors (age, sex, race, education level, employment status, household income, primary caregiver), clinical factors (pre-existing comorbidities, severity of Covid-19, duration of symptoms, presence of long Covid), and care-related factors (hospitalization, ICU admission, need for ventilatory support).

It is recognized that, in cross-sectional studies, the distinction between confounders and mediators can be complex, since some variables (such as income) may act through indirect causal pathways. Multivariate analyses were designed to control factors considered direct confounders, as specified in the model construction, as well as to explore the observed associations within the limitations inherent to the cross-sectional design.

A Poisson regression on a logarithmic scale with robust variance was performed. For the construction of the final multivariate models, variables that showed p < 0.20 in the bivariate analysis were included, in addition to those considered potential *a priori* confounders based on the literature and epidemiological relevance (age, race, sex, and education level), regardless of their initial significance. This approach sought to mitigate the limitations of purely stepwise models, prioritizing theoretical consistency and control of known confounding factors.

The final models were constructed using two criteria: Model 1, which included all functional variables—BADL (Katz), IADL (Lawton), and functional implications (WHODAS)—and Model 2, which included only functional implications (WHODAS).

For the present analysis, only fully completed electronic questionnaires for the variables of interest were considered (complete-case analysis); no imputation methods were used for missing data.

## RESULTS

Based on the 337,420 cases, 775 individuals participated in this study, of whom 395 were classified as mild cases of Covid-19 (50.9%) and 380 as hospitalized cases (49.0%). Among these, 240 (63.1%) progressed to severe disease, presenting with respiratory distress and desaturation; 14 (3.8%) presented with diffuse abdominal pain and gastrointestinal symptoms; and 126 (33.1%) progressed to a critical condition, requiring admission to the ICU due to worsening of the underlying condition, respiratory failure, hypotension and/or significant arrhythmias, poor peripheral perfusion, or a fever lasting more than 48 hours, along with altered mental status or lethargy (Figure).

**Figure:**
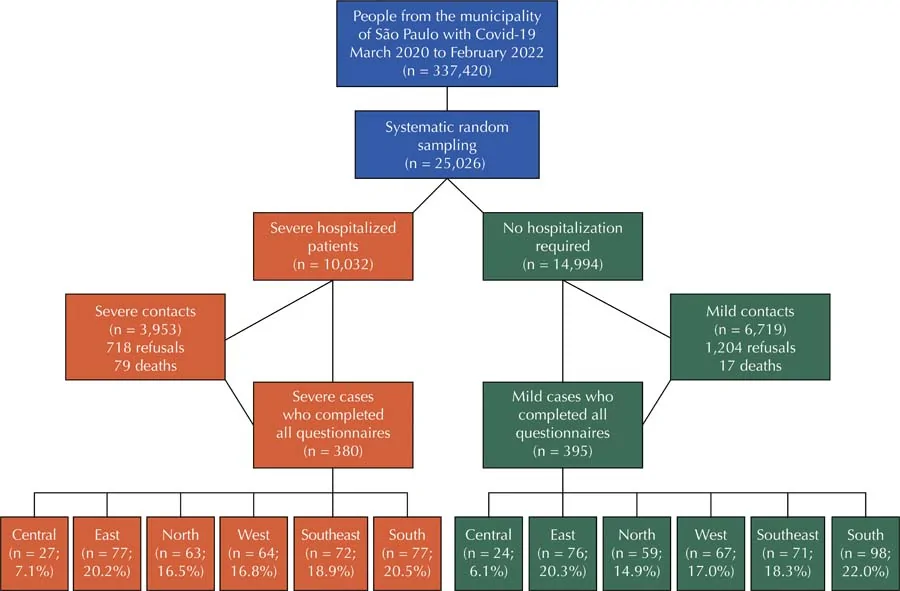
Study flowchart.

The mean time from the onset of the first symptoms, as recorded in the notification, until study participation was 29.5 months (median: 29 months; interquartile range: 11 months). The mean age of the study participants was 49 years (18–93), and 435 (56.1%) were female.

As illustrated in the Figure, the final sample of 775 participants consists of individuals who accepted and completed the questionnaires following an initial invitation sent to 25,026 randomly selected individuals. Due to refusals and loss to follow-up, the sample of respondents can be considered a convenience sample, which constitutes a limitation discussed below. However, the sample size achieved was sufficient to ensure 99.0% statistical power for the outcomes studied.

Regarding employment, 295 (72.6%) of the mild cases and 263 (69.2%) of the severe cases reported having paid work before Covid-19. After infection, 281 (71.1%) of the mild cases and 248 (65.2%) of the severe cases reported remaining in the same job. Regarding employment status, among mild cases, 231 (58.4%) had formal employment and 64 (16.2%) had informal employment; among severe cases, 188 (49.4%) had formal employment and 38 (10.0%) had informal employment.

Among severe cases, 329 (75.6%) workers had long Covid, while among mild cases, 86 (19.7%) had the same condition. Of these, 121 (27.5%) of the severe cases reported reduced work performance, while 100 (22.7%) of the mild cases reported the same condition.

Tables 1A and 1B present the associations between sociodemographic, clinical, care-related, and functional characteristics and reduced work performance and difficulty returning to work, respectively.

The prevalence of reduced work performance was 43.9% (95%CI 39.7–48.2), with 48.6% (95%CI 42.4–54.8) in severe cases and 39.6% (95%CI 33.9–45.5) in mild cases. Difficulty returning to work was observed in 22.2% (95%CI 18.8–25.9) of individuals who had Covid-19, with 30.1% (95%CI 24.6–36.1) in severe cases and 15.0% (95%CI 11.0–19.7) in mild cases. Regarding lower work performance, severe cases had a prevalence ratio of 1.22 compared to mild cases (95%CI 1.01–1.48). As for difficulty returning to work, severe cases had a prevalence ratio twice that of mild cases (95%CI 1.43–2.79). Long Covid, defined as the persistence of two or more symptoms for more than four weeks after infection, had a prevalence ratio of 1.15 for reduced work performance (95%CI 1.04–1.46).

**Graph 1:**
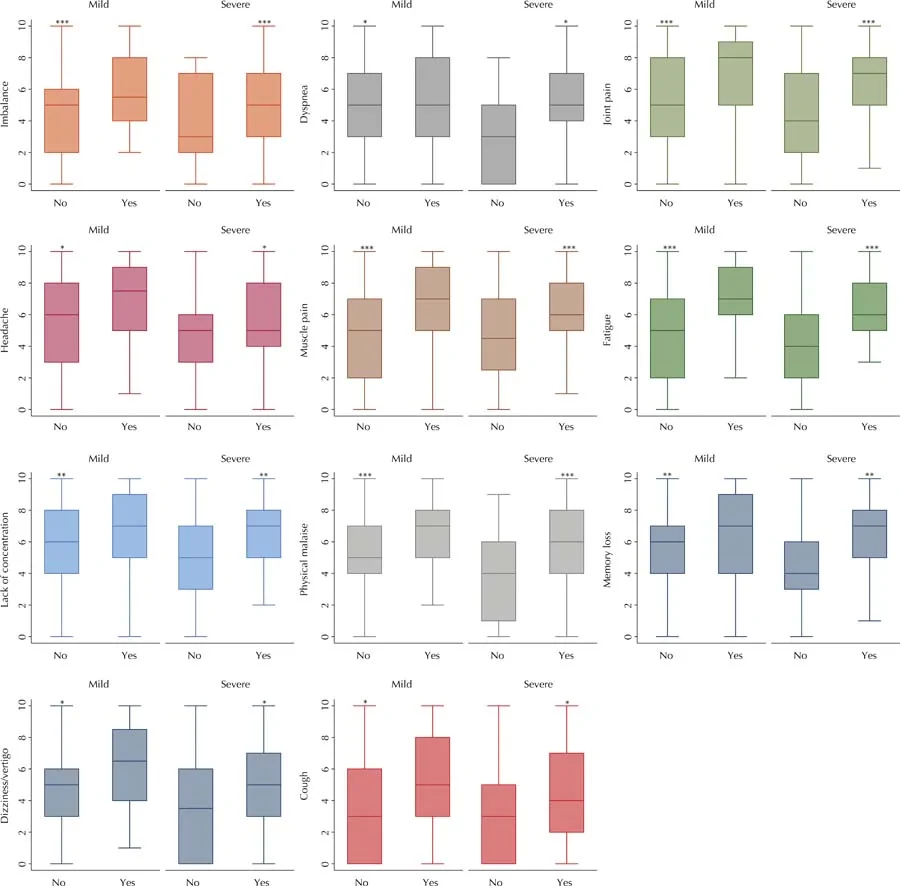
Severity of persistent post-Covid-19 symptoms according to work performance and disease severity.

Note: boxplots of post-Covid-19 symptom intensity according to work performance and disease severity. The vertical axis represents the self-reported intensity of each symptom (scale from 0 to 10), while the horizontal axis indicates the presence of an impact on work performance, categorized as “yes” (there was an influence) or “no” (there was no influence), stratified according to the severity of Covid-19. A two-way ANOVA demonstrated a significant effect of work performance (p < 0.05), with no interaction with severity.

Graph 1 shows that poorer work performance was significantly associated with increased intensity of all symptoms assessed, including joint pain (F = 31.77; p < 0.001), muscle pain (F = 26.86; p < 0.001), dizziness/vertigo (F = 12.09; p = 0.0006), headache (F = 10.26; p = 0.0016), dyspnea (F = 11.52; p = 0.0008), fatigue (F = 72.15; p < 0.001), attention deficit (F = 16.60; p < 0.001), memory loss (F = 19.74; p < 0.001), physical malaise (F = 41.19; p < 0.001), cough (F = 10.43; p = 0.0015), and loss of balance (F = 5.79; p = 0.017).

*p < 0.05.

**p < 0.01.

***p < 0.001.

The severity of Covid-19 was significantly associated with dizziness (F = 10.77; p = 0.0012), headache (F = 6.58; p = 0.011), lack of concentration (F = 5.05; p = 0.025), memory loss (F = 5.22; p = 0.023), dyspnea (F = 6.50; p = 0.011), fatigue (F = 8.92; p = 0.003), and physical malaise (F = 8.48; p = 0.0039), but not with joint pain, muscle pain, cough, or balance (p > 0.05).

There was no significant interaction between work performance and disease severity for any of the symptoms assessed (p > 0.05), indicating that the impact of work performance on symptom intensity occurs independently of the severity of the acute phase of the infection.

**Graph 2:**
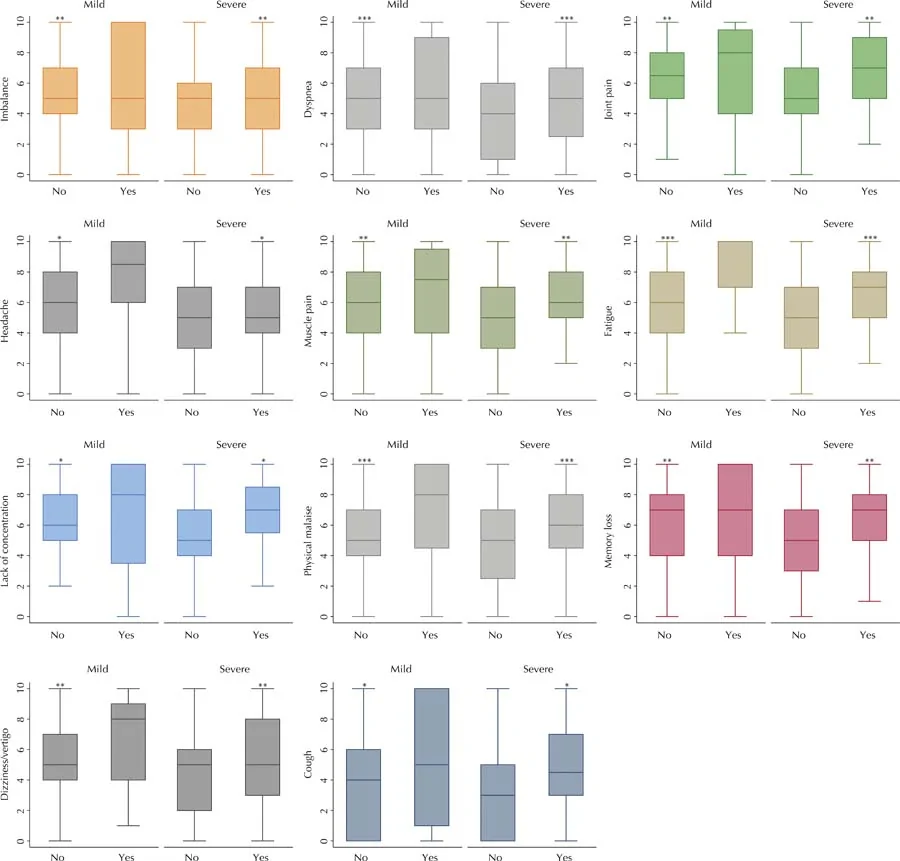
Intensity of persistent post-Covid-19 symptoms according to difficulty returning to work and disease severity.

Note: boxplots of the intensity of post-Covid-19 symptoms according to difficulty returning to work and disease severity. The vertical axis represents the self-reported intensity of each symptom (numeric scale from 0 to 10). The horizontal axis indicates the presence of difficulty returning to work, categorized as “yes” (with difficulty) or “no” (without difficulty), stratified according to the severity of Covid-19. A two-way ANOVA demonstrated a significant effect of difficulty returning to work (p < 0.05), with no interaction with severity.

Graph 2 shows that difficulty returning to work was significantly associated with increased intensity of several persistent symptoms, including joint pain (F = 5.94; p = 0.015), muscle pain (F = 5.83; p = 0.016), dyspnea (F = 5.79; p = 0.017), fatigue (F = 30.55; p < 0.001), lack of concentration (F = 6.96; p = 0.009), memory loss (F = 5.37; p = 0.021), cough (F = 6.84; p = 0.010), and physical malaise (F = 17.65; p < 0.001).

*p < 0.05.

**p < 0.01.

***p < 0.001.

The severity of Covid-19 was significantly associated with dizziness/vertigo (F = 10.28; p = 0.002), headache (F = 9.08; p = 0.003), dyspnea (F = 5.62; p = 0.019), fatigue (F = 9.74; p = 0.002), physical malaise (F = 5.51; p = 0.020), and imbalance (F = 3.95; p = 0.049).

No significant interaction was observed between difficulty returning to work and disease severity for any of the symptoms assessed (p > 0.05), indicating that the effect of functional limitation occurs independently of the severity of the acute phase.

Table 2 presents the multivariate Poisson regression models for reduced work performance. It can be observed that, regardless of severity and time elapsed since infection, there was no association with reduced work performance. However, the following variables were associated with reduced work performance: “being the primary caregiver” (PR = 1.55; 95%CI 1.05–2.29), “moderate functional implications” (OR = 2.69; 95%CI 1.44–5.01), and “severe” (OR = 4.21; 95%CI 2.22–7.95) were associated with reduced work performance (Model 1).

**Table 2:** Multivariate logistic regression for reduced work performance following Covid-19.

Independent variable		Reduced work performance			
		Model 1		Model 2	
		PR (95%CI)	p-value	PR (95%CI)	p-value
Age	18–59 years	1		1	
	**>** 60 years	1.22 (0.82–1.81)	0.317	**2.20 (1.27–3.80)**	**0.036**
Gender	Male	1		1	
	Female	0.98 (0.72–1.32)	0.902	0.89 (0.69–1.15)	0.396
Race	White	1		1	
	Black/Brown	0.96 (0.56–1.63)	0.354	0.96 (0.32–1.97)	0.873
	Other	0.34 (0.08–1.39)	0.959	0.38 (0.76–9.11)	0.181
Education	Elementary	1		1	
	High school	0.87 (0.54–1.40)	0.574	0.96 (0.22–1.24)	0.877
	Higher	1.07 (0.68–1.67)	0.749	1.14 (0.76–1.70)	0.511
Severity of Covid-19	Mild	1		1	
	Severe	0.84 (0.61–1.16)	0.303	1.01 (0.78–1.28)	0.960
Duration of Covid-19 symptoms (months)	15–29	1		1	
	30–50	1.22 (0.92–1.62)	0.166	1.15 (0.89–1.49)	0.281
Long Covid	Yes	1.75 (0.48–6.40)	0.395	1.71 (0.45–6.48)	0.428
Income	R$ 500.00–R$ 2,000.00	1		1	
	R$ 2,000.00–R$ 5,000.00	1.11 (0.72–1.71)	0.624	1.30 (0.74–1.59)	0.662
	R$ 5,000.00–R$ 10,000.00	1.06 (0.64–1.75)	0.794	0.97 (0.60–1.61)	0.850
	> R$ 10,000.00	0.94 (0.55–1.59)	0.829	1.20 (0.58–1.49)	0.780
Primary caregiver		1.55 (1.05–2.29**)**	**0.024**	1.30 (1.02–1.66)	**0.028**
Comorbidity Pre-existing	Hypertension	1.31 (0.96–1.79)	0.081	1.30 (0.97–1.75)	0.075
	COPD	1.06 (0.71–1.59)	0.759	0.97 (0.68–1.37)	0.287
	Diabetes	1.15 (0.83–1.59)	0.388	1.20 (0.90–1.60)	0.865
Sarcopenia	Sarcopenic	1.06 (0.83–1.36)	0.597		
ABVD (Katz)	Independent	1			
	Partially dependent	1.05 (0.73–1.51)	0.761		
	Totally dependent	1.14 (0.78–1.67)	0.487		
AIVD (Lawton)	Dependent	1.10 (0.52–2.33)	0.789		
Functional impairment (WHODAS)	Mild	1.46 (0.74–2.90)	0.260	1.90 (0.99–3.67)	0.053
	Moderate	2.69 (1.44–5.01)	**0.002**	2.99 (1.60–5.58)	**0.001**
	Severe	4.21 (2.22–7.95)	**< 0.001**	4.88 (2.59–9.19)	**< 0.001**

PR: prevalence ratio; 95%CI: 95% confidence interval; COPD: chronic obstructive pulmonary disease; BADL: activities of daily living immediately after Covid-19; IADL: instrumental activities of daily living immediately after Covid-19; WHODAS: World Health Organization Disability Assessment Schedule.

Note: Model 1 was constructed using all functional variables: BADL (Katz); IADL (Lawton); and functional implications (WHODAS), while Model 2 was constructed using only functional implications (WHODAS) to improve the interpretation and understanding of the factors influencing the outcomes. P-values in bold indicate factors significantly associated with the outcome of lower work performance (p < 0.05).

In Model 2, which includes only WHODAS domains as functional variables, advanced age (OR = 2.20; 95%CI 1.27–3.80) and moderate and severe functional impairments were most strongly associated with lower work performance: (OR = 2.99; 95%CI 1.60–5.58); p = 0.001 and (OR= 4.88; 95%CI 2.59–9.19; p = < 0.001), respectively.

Table 3 presents two multivariate Poisson regression models for the factors associated with sequelae that hindered return to work. The main association observed was with severe functional impairment (PR = 3.91; 95%CI 1.72–8.89; p = 0.001). In addition, factors such as advanced age and sarcopenia were also associated with difficulty returning to work.

**Table 3:** Multivariate logistic regression of difficulty returning to work after Covid-19.

Independent variable		Difficulty returning to work			
		Model 1		Model 2	
		PR (95%CI)	p-value	PR (95%CI)	p-value
Age	18–59 years	1		1	
	> 60 years	2.18 (1.05–4.49)	0.034	2.20 (1.27–3.80)	**0.036**
Gender	Male	1		1	
	Female	1.22 (0.62–2.39)	0.548	1.05 (0.65–1.78)	0.815
Race	White	1		1	
	Black/Brown	0.38 (0.92–2.34)	0.354	0.80 (0.32–1.97)	0.633
	Other	1.24 (0.68–12.7)	0.959	0.83 (0.76–9.11)	0.883
Education	Elementary	1		1	
	High school	0.66 (0.17–2.43)	0.533	0.52 (0.22–1.24)	0.143
	High	1.07 (0.36–3.16)	0.895	0.78 (0.40–1.53)	0.486
Severity of Covid-19	Mild	1		1	
	Severe	1.68 (0.76–3.74)	0.197	1.49 (0.81–2.73)	0.193
Duration of Covid-19 symptoms (months)	15–29	1		1	
	30–50	1.07 (0.56–2.04)	0.829	1.17 (0.63–2.07)	0.527
Long Covid		0.53 (0.12–2.26)	0.393	0.77 (0.19–3.13)	0.724
Income	R$ 500.00–R$ 2,000.00	1		1	
	R$ 2,000.00–R$ 5,000.00	1.10 (0.48–2.55)	0.806	1.14 (0.58–2.22)	0.690
	R$ 5,000.00–R$ 10,000.00	0.70 (0.23–2.10)	0.533	0.86 (0.37–2.01)	0.731
	> R$ 10,000	0.72 (0.26–1.99)	0.534	0.71 (0.30–1.67)	0.443
Being the primary caregiver		1.24 (0.59–2.57)	0.563	1.01 (0.75–1.76)	0.947
Pre-existing comorbidity	Hypertension	1.34 (1.01–3.52)	0.286	1.16 (0.72–1.87)	0.532
	COPD	0.97 (0.78–2.29)	0.717	1.21 (0.71–2.08)	0.467
	Diabetes	0.83 (0.46–2.02)	0.620	1.35 (0.85–2.14)	0.194
Sarcopenia	Sarcopenic	1.96 (1.08–3.55)	0.026	1.95 (1.18–3.24)	**0.009**
ABVD (Katz) impairment	Independent	1			
	Partially dependent	1.02 (0.43–2.39)	0.963		
	Totally dependent	1.06 (0.57–1.97)	0.854		
AIVD (Lawton)	Dependent	2.36 (0.71–7.79)	0.156		
Functional impairment (WHODAS)	Mild	1.24 (0.40–3.83)	0.697	1.34 (0.53–3.37)	0.529
	Moderate	1.97 (0.67–5.78)	0.212	2.15 (0.89–5.20)	0.087
	Severe	4.33 (1.58–11.8)	0.004	3.91 (1.72–8.89)	**0.001**

PR: prevalence ratio; 95%CI: 95% confidence interval; COPD: chronic obstructive pulmonary disease; BADL: activities of daily living immediately after Covid-19; IADL: instrumental activities of daily living immediately after Covid-19; WHODAS: World Health Organization Disability Assessment Schedule.

Note: Model 1 was constructed using all functional variables: BADL (Katz); IADL (Lawton); and functional implications (WHODAS), while Model 2 included only SARC-F and WHODAS to improve the interpretation and understanding of the factors influencing the outcomes. P-values in bold indicate factors associated with the outcome of difficulty returning to work (p < 0.05).

## DISCUSSION

This study shows that sociodemographic, clinical, and functional factors are associated with changes in work activities, both in terms of performance and return to work. Factors such as advanced age, being the primary caregiver, and having moderate or severe functional impairment were associated with lower work performance. Furthermore, advanced age, sarcopenia, and severe functional impairment were associated with an inability to return to work.

According to the findings of this study, the time between the onset of symptoms and the date the survey was completed was not significantly associated with lower work performance or with return to work. However, as age increases (> 60 years), the prevalence ratio reaches 2.20 times for this population. These results corroborate those found in the studies by Chen et al.^22^ and Westerlind et al.^9^, in which advanced age was a predictor of worse health outcomes and difficulty or a prolonged time to return to work. One hypothesis for this phenomenon is immunosenescence, a process of gradual deterioration of the immune system resulting from aging, which leads to greater vulnerability to infections and a less effective response to vaccinations^23^.

It was expected that the severity of Covid-19 would be associated with poorer work performance and difficulty returning to work; however, this was not observed.

Long Covid is more common among adult women, those with type 2 diabetes mellitus, older adults, individuals with preexisting comorbidities, those with an incomplete Covid-19 vaccination series, low-income individuals, those with lower educational attainment, and those who experienced a more severe acute phase requiring ICU admission^24^. Azevedo et al.^3^, in turn, showed in their cohort study that, although most patients returned to work after discharge, approximately 70% presented with symptoms related to long Covid between six and nine months after infection. Our cross-sectional findings, although they do not allow for the same longitudinal inference, are consistent with the persistence of symptoms observed in the literature.

This study identified the prevalence of 75.6% of workers with long Covid among severe cases and 19.7% among those with mild cases. In this study, long Covid, regardless of severity or duration of symptoms, was not significantly associated with reduced work performance or difficulty returning to work. However, the cross-sectional results indicate that, at the time of data collection, many workers who reported long Covid also faced difficulties in work performance or in returning to work, which highlights the complexity of the interactions between health status and the work environment.

It is important to recognize that this prevalence may have been influenced by selection bias, since individuals with persistent symptoms (long Covid) may have been more likely to participate in the study. Consequently, this variable should be interpreted with caution, understanding it as a characteristic of the sample that may be associated with the outcomes, without allowing for direct causal inferences.

Although most participants had an income exceeding R$ 2,000.00 and a high level of education (completed higher education), a significant association was observed between being the primary caregiver and lower work performance. This finding corroborates the study by Bejot et al.^28^, which highlights the social pressure exerted on a single family member responsible for supporting the household. This concentrated financial responsibility can impair work performance and contribute to a cumulative disadvantage, leading to financial difficulties and reduced access to health care in the long term^29^.

Regarding the long-term effects of Covid-19, early interventions have been associated with improved recovery and quality of life^30^. Home-based or outpatient rehabilitation may be necessary to treat these long-term effects, and the improvement of persistent symptoms, such as fatigue, can directly influence a faster return to work^31^.

The study by Van Wambeke et al*.*^32^ found that comorbidities such as systemic hypertension, diabetes mellitus, and chronic obstructive pulmonary disease play a significant role in patients’ functional decline, directly affecting work performance and the time required to return to work after Covid-19, which differs from this study, in which preexisting comorbidities (hypertension, chronic obstructive pulmonary disease, and diabetes) were not associated with reduced work performance or difficulty returning to work.

Regarding possible post-infection consequences, Morley^33^ notes that Covid-19 survivors may experience a decline in muscle function, with an increased risk of acute sarcopenia. In critically ill patients who are hospitalized and on mechanical ventilation, the reduction in muscle mass can reach up to 2% per day^34^.

Other factors contributing to sarcopenia include social isolation and the increase in remote work, which were adopted as measures to contain the spread of the virus^34^. The study by Silva et al.^35^ demonstrated a 26.0% increase in physical inactivity during this period. In the present study, sarcopenia was present in 22.7% of participants with reduced work performance and in 26.6% who had difficulty returning to work, and it was associated with up to a 1.96-fold increased risk of difficulty returning to work.

In addition to post-Covid sequelae, the association between moderate to severe functional impairment and reduced work performance, as well as severe functional impairment associated with difficulty returning to work, was highlighted. Vanichkachorn et al.^36^, in a cohort study, describe long Covid as a condition that can persist for months after the initial infection, resulting in significant functional impairment. These clinical and functional factors can negatively impact functional capacity and quality of life, in addition to hindering a return to work. Thus, the present findings complement this perspective by raising the hypothesis of an association between functional limitations, reduced work performance, and difficulty returning to work.

Therefore, this study highlights the need to broaden our perspective on individuals affected by the sequelae of Covid-19, considering not only physical aspects but also the social, economic, and functional factors associated with reduced work performance and difficulty returning to work. Regardless of the severity and duration of symptoms, associations with impairments in work activities were observed. Timely interventions, through intersectoral actions in health, work, and interdisciplinary rehabilitation, can facilitate both a return to work and improved work performance.

In terms of limitations, this study was conducted using a self-administered questionnaire between 15 and 50 months after the initial Covid-19 infection. Consequently, memory bias may have occurred. We acknowledge that a possible selection bias occurred due to the high non-response rate, stemming from participants’ reluctance to answer the survey over the phone. An additional limitation is that, due to the self-administered nature of the questionnaire and the inability to compare sociodemographic or clinical characteristics between participants and non-respondents using available secondary data, it was not possible to investigate potential selection biases related to these basic characteristics (age, sex, education level) that could have influenced the results. This lack of information may limit the generalizability of our findings.

Given the uncertainties regarding the long-term impacts of the Covid-19 pandemic, we recommend conducting longitudinal studies to investigate the social, clinical, care-related, and functional effects, as well as their relationship to performance and return to work, thereby enabling the establishment of more robust causal relationships.

## CONCLUSION

People who had both severe and mild forms of Covid-19 returned to work; however, nearly half exhibited reduced work performance. The proportion of impaired work performance was higher among severe cases, and long Covid was not associated with reduced work performance or difficulty returning to work.

Factors such as advanced age and moderate-to-severe functional impairment were associated with reduced work performance. On the other hand, difficulty returning to work was primarily associated with advanced age, sarcopenia, and severe functional impairment.

Thus, the importance of intersectoral collaboration between health and labor sectors is highlighted, with an emphasis on the individual and collective care needs of these workers to optimize functional capacity and improve quality of life at work.

**Table 1:** Sociodemographic, clinical, care-related, and functional characteristics associated with work performance and return-to-work.

Characteristic	Lower work performance			No influence on work performance			p-value^a^
	Severe	Mild	Total	Severe	Mild	Total	
	n (%)	n (%)		n (%)	n (%)		
**Sociodemographic**							
Age in years							0.659
18–59	69 (47.5)	95 (95.9)	164 (79.6)	76 (68.5)	132 (91.0)	208 (81.2)	
> 60	38 (52.5)	4 (4.1)	42 (20.4)	35 (31.5)	13 (9.0)	48 (18.8)	
Gender							0.405
Male	70 (56.0)	36 (31.8)	106 (43.9)	86 (63.7)	60 (34.9)	146 (47.5)	
Female	58 (44.0)	77 (68.2)	135 (56.1)	49 (36.3)	112 (65.1)	161 (52.5)	
Race							0.888
White	84 (66.7)	55 (49.5)	139 (58.6)	94 (70.1)	90 (53.2)	184 (60.7)	
Black/Brown	15 (11.9)	19 (17.1)	34 (14.3)	11 (8.2)	31 (18.3)	42 (13.8)	
Other	27 (21.4)	37 (33.4)	64 (27.1)	29 (21.7)	48 (28.5)	77 (25.5)	
Marital Status							0.757
Single	19 (15.4)	45 (39.8)	64 (27.0)	26 (19.5)	66 (59.5)	92 (30.7)	
Married	85 (68.5)	53 (46.9)	138 (58.2)	81 (61.0)	80 (60.2)	161 (53.7)	
Divorced	14 (11.3)	14 (12.4)	28 (11.8)	19 (14.3)	19 (57.6)	38 (12.7)	
Widower	6 (4.8)	1 (0.9)	7 (3.0)	7 (5.2)	2 (66.6)	9 (2.9)	
Education							0.551
Elementary school	12 (17.1)	9 (11.5)	21 (14.1)	9 (9.9)	8 (6.7)	17 (8.1)	
High school	12 (17.1)	28 (34.6)	40 (27.0)	12 (13.2)	44 (37.0)	56 (27.0)	
Higher education	46 (65.8)	41 (52.5)	87 (58.7)	70 (76.9)	67 (56.3)	137 (64.9)	
Employment status							0.251
Formal	73 (60.8)	74 (66.0)	147 (63.3)	96 (72.7)	121 (68.7)	217 (71.6)	
Informal	16 (13.3)	15 (13.4)	31 (12.8)	7 (5.3)	21 (11.9)	28 (9.2)	
Income							**0.006**^a^
Up to R$ 2,000.00	12 (11.2)	40 (37.4)	52 (24.3)	12 (10.2)	32 (20.4)	44 (16.0)	
R$ 2,000.00–R$ 5,000.00	42 (39.2)	44 (41.1)	86 (40.2)	26 (22.0)	66 (42.0)	92 (33.4)	
R$ 5,000.00–R$ 10,000.00	22 (20.6)	13 (12.1)	35 (16.3)	35 (29.7)	32 (20.4)	67 (24.4)	
> R$ 10,000.00	31 (29.0)	10 (9.4)	41 (19.2)	45 (38.1)	27 (17.2)	72 (26.2)	
Primary caregiver	99 (34.6)	86 (30.0)	185 (64.6)	36 (12.5)	65 (22.9)	101 (35.4)	**0.013**^a^
**Clinical**							
Comorbidity							
Hypertension	42 (26.4)	25 (28.0)	67 (27.0)	30 (24.0)	25 (25.5)	55 (24.7)	**0.006**^a^
Diabetes	27 (17.0)	9 (10.1)	36 (14.5)	21 (16.8)	10 (10.2)	31 (13.9)	**0.006**^a^
Obesity	67 (42.1)	38 (42.7)	105 (42.3)	59 (47.2)	55 (56.1)	114 (51.1)	0.132
COPD	23 (14.5)	17 (19.2)	40 (16.2)	15 (12.0)	8 (8.2)	23 (10.3)	**0.001**^a^
Duration of symptoms (months)							0.515
15–29	52 (21.5)	70 (29.0)	122 (50.5)	49 (16.4)	115 (36.0)	156 (52.4)	
30–50	76 (31.5)	43 (18.0)	119 (49.5)	86 (28.8)	57 (18.8)	142 (47.6)	
Long Covid	121 (27.5)	100 (22.7)	221 (50.2)	105 (23.8)	114 (26.0)	219 (49.8)	**0.012**^a^
**Healthcare**							
Hospitalized	127 (73.8)	**-**	127 (73.8)	135 (76.3)	**-**	135 (76.3)	0.304
ICU	45 (26.4)	**-**	45 (26.4)	42 (23.7)	**-**	42 (23.7)	0.414
Ventilatory support					**-**	**-**	0.076
Invasive	12 (15.6)	**-**	12 (15.6)	7 (9.2)	**-**	7 (9.2)	
Non-invasive	65 (84.4)	**-**	65 (84.4)	69 (90.8)	**-**	69 (90.8)	
**Functional**							
Basic activities of daily living (immediately after Covid-19)							**< 0.001**^a^
Full function	50 (52.1)	90 (83.3)	140 (68.6)	95 (76.0)	144 (92.4)	239 (85.0)	
Partially dependent	14 (14.6)	12 (11.1)	26 (12.7)	17 (13.6)	6 (3.8)	23 (8.2)	
Totally dependent	32 (33.3)	6 (5.6)	38 (18.7)	13 (10.4)	6 (3.8)	19 (6.8)	
Instrumental activities of daily living (immediately after Covid-19)							**0.010**^a^
Independent	111 (81.7)	102 (98.0)	213 (88.7)	167 (93.3)	123 (96.8)	290 (94.8)	
Dependent	25 (18.3)	2 (2.0)	27 (11.3)	12 (6.7)	4 (3.2)	16 (5.2)	
Sarcopenia							**< 0.001**^a^
Normal weight	90 (78.2)	77 (76.2)	167 (77.3)	124 (96.1)	138 (95.2)	262 (95.6)	
Sarcopenic	25 (21.8)	24 (23.8)	49 (22.7)	5 (3.9)	7 (4.8)	12 (4.4)	
Functional impairment in WHODAS							**< 0.001**^a^
Mild	9 (7.6)	8 (7.7)	17 (7.6)	66 (50.8)	53 (37.1)	119 (42.0)	
Moderate	63 (52.9)	49 (47.1)	112 (50.2)	55 (42.3)	89 (62.2)	144 (50.8)	
Severe	47 (39.5)	47 (45.2)	94 (42.2)	9 (6.9)	11 (0.7)	20 (7.2)	
**Characteristic**	**Difficulty returning to work**			**No difficulty returning to work**			**p-value^a^**
	**Severe**	**Mild**	**Total**	**Severe**	**Mild**	**Total**	
	**n (%)**	**n (%)**		**n (%)**	**n (%)**		
**Sociodemographic**							
Age in years							**0.002**^a^
18–59	35 (55.5)	33 (94.3)	68 (69.4)	109 (70.8)	195 (92.8)	314 (83.9)	
> 60	28 (44.5)	2 (5.7)	30 (30.6)	45 (29.2)	15 (7.2)	60 (16.1)	
Gender							0.523
Male	42 (53.2)	11 (25.6)	53 (43.5)	111 (91.0)	85 (35.0)	196 (53.7)	
Female	37 (46.8)	32 (74.4)	69 (56.5)	11 (9.0)	158 (65.0)	169 (46.3)	
Race							0.626
White	49 (83.0)	19 (79.2)	68 (81.9)	128 (88.9)	125 (73.1)	253 (80.6)	
Black/Brown	10 (17.0)	5 (20.8)	15 (18.1)	16 (11.1)	45 (26.3)	61 (19.4)	
Other	**-**	**-**	**-**	**-**	1 (0.6)	**-**	
Marital Status							0.809
Single	13 (17.1)	20 (46.5)	33 (27.7)	33 (18.3)	93 (39.1)	126 (30.1)	
Married	48 (63.1)	19 (44.2)	67 (56.3)	117 (65.0)	114 (47.9)	231 (55.3)	
Divorced	11 (14.5)	3 (6.9)	14 (11.7)	21 (11.6)	29 (12.2)	50 (12.0)	
Widower	4 (5.3)	1 (2.4)	5 (4.3)	9 (5.1)	2 (0.8)	11 (2.6)	
Education							**0.013**^a^
Elementary	12 (25.0)	3 (10.3)	15 (19.5)	9 (7.4)	14 (8.3)	23 (7.9)	
High School	8 (16.6)	12 (41.4)	20 (26.0)	25 (20.7)	61 (36.1)	86 (29.6)	
Higher education	28 (58.4)	14 (48.3)	42 (54.5)	87 (71.9)	94 (55.6)	181 (62.5)	
Employment Status							**< 0.001**^a^
Formal	36 (48.6)	22 (52.4)	58 (50.0)	133 (75.1)	175 (72.3)	312 (73.7)	
Informal	10 (13.5)	6 (14.3)	16 (13.8)	13 (7.3)	30 (12.4)	43 (10.2)	
Income							**< 0.001**^a^
Up to R$2,000.00	11 (17.7)	18 (45.0)	29 (20.6)	12 (7.4)	55 (24.5)	67 (17.8)	
R$ 2,000.00–R$ 5,000.00	26 (41.9)	16 (40.0)	42 (45.6)	43 (26.4)	94 (42.0)	137 (36.3)	
R$ 5,000.00–R$ 10,000.00	12 (19.3)	4 (10.0)	16 (17.4)	45 (27.6)	40 (17.8)	85 (22.5)	
> R$ 10,000.00	13 (21.1)	2 (5.0)	15 (16.4)	63 (38.6)	35 (15.7)	88 (23.4)	
Primary caregiver	56 (26.4)	33 (15.5)	89 (41.9)	42 (19.8)	81 (38.3)	123 (58.1)	0.718
**Clinical**							
Comorbidity							
Hypertension	24 (24.7)	38 (56.7)	62 (37.8)	12 (7.9)	48 (30.4)	60 (19.3)	**0.029**^a^
Diabetes	19 (19.6)	6 (8.9)	25 (15.2)	30 (19.7)	14 (8.8)	44 (14.2)	**0.003**^a^
Obesity	40 (41.2)	15 (22.4)	55 (33.5)	86 (56.6)	79 (50.0)	165 (53.2)	0.167
COPD	14 (14.4)	8 (12.0)	22 (13.5)	24 (15.8)	17 (10.8)	41 (13.3)	**0.010**^a^
Duration of symptoms (months)							0.892
15–29	38 (31.1)	27 (22.1)	65 (53.2)	63 (27.8)	161 (25.2)	224 (53.0)	
30–50	41 (33.6)	16 (13.2)	57 (46.8)	120 (28.0)	82 (19.0)	202 (47.0)	
Long Covid	75 (17.0)	36 (8.2)	111 (25.2)	150 (34.1)	179 (30.7)	329 (74.8)	0.133
**Healthcare**							
Hospitalized	79 (71.8)	**-**	79 (71.8)	182 (61.3)	**-**	182 (61.3)	0.510
ICU	31 (28.2)	**-**	31 (28.2)	115 (38.7)	**-**	115 (38.7)	0.250
Ventilatory support							**0.001**^a^
Invasive	13 (25.5)	**-**	13 (25.5)	6 (5.9)	**-**	6 (5.9)	
Non-invasive	38 (74.5)	**-**	38 (74.5)	96 (94.1)	**-**	96 (94.1)	
**Functional**							
Basic activities of daily living (immediately after Covid-19)							**< 0.001**^a^
Full function	25 (47.2)	30 (75.0)	55 (61.1)	119 (71.2)	203 (91.0)	322 (82.3)	
Partially dependent	4 (7.5)	6 (15.0)	10 (11.1)	27 (16.2)	13 (5.8)	40 (10.2)	
Totally dependent	24 (45.3)	4 (10.0)	28 (27.8)	21 (12.6)	8 (3.2)	29 (7.5)	
Instrumental activities of daily living (immediately after Covid-19)							**< 0.001**^a^
Independent	52 (69.3)	36 (92.3)	88 (77.2)	165 (92.2)	221 (98.7)	386 (95.8)	
Dependent	23 (30.7)	3 (6.7)	26 (22.8)	14 (7.8)	3 (1.3)	17 (4.2)	
Sarcopenia							**< 0.001**^a^
Eutrophic	55 (78.5)	25 (64.1)	80 (73.4)	158 (91.3)	190 (91.8)	248 (88.6)	
Sarcopenic	15 (21.5)	14 (35.9)	29 (26.6)	15 (8.7)	17 (8.2)	32 (11.4)	
Functional impairment in WHODAS							**< 0.001**^a^
Mild	7 (9.6)	2 (5.0)	9 (8.0)	67 (38.3)	58 (26.7)	125 (31.9)	
Moderate	37 (50.7)	13 (32.5)	50 (44.2)	80 (45.7)	126 (58.1)	206 (52.5)	
Severe	29 (39.5)	25 (62.5)	54 (47.8)	28 (16.0)	33 (15.2)	61 (15.6)	

ICU: intensive care unit; WHODAS: World Health Organization Disability Assessment Schedule; COPD: chronic obstructive pulmonary disease.

Note: values with a statistically significant association are highlighted in bold.

^a^ Statistically significant association (p < 0.05) between sociodemographic, clinical, care-related, and functional factors and reduced work performance, as well as barriers to returning to work, compared to the group with no influence on work performance and no barriers to returning to work (chi-square test).
