# Supplementary material for: Functional impact of long Covid on work performance and return to work: a cross-sectional study
Source: Rev Saude Publica. 2026 Aug 7;60:e35. doi: 10.11606/s1518-8787.2026060007097 (PMC13480950; doi:10.11606/s1518-8787.2026060007097)
Supplement: Translation

# Translation: Impacto funcional da covid longa no desempenho laboral e no retorno ao trabalho: um estudo transversal

**Authors:** Fabio Cavalcanti Freitas, Vivian Cintra Sousa, Daniella de Melo, Diêgo Araújo de Moraes Ramos, Gabriela Sayuri Ochiai, Daniel Kolisch, Julia Costa Guimarães, Larissa Gomes da Silva, Erika Christina Gouveia e Silva, José Eduardo Pompeu, Ana Carolina Basso Schmitt

**Affiliations:** I Brasil Universidade de São Paulo. Faculdade de Medicina. Departamento de Fisioterapia, Fonoaudiologia e Terapia Ocupacional. São Paulo, SP, Brasil

**Keywords:** Síndrome de Pós-COVID-19 Aguda, Estado Funcional, Desempenho Laboral, Retorno ao Trabalho

## Abstract

**OBJETIVO:**

Avaliar a associação entre a covid longa e os fatores socioeconômicos, clínicos, assistenciais e funcionais com o menor desempenho laboral e dificuldade no retorno ao trabalho.

**MÉTODOS:**

Estudo transversal com indivíduos de São Paulo ≥ 18 anos, com covid longa (77,8%), com coleta entre abril de 2023 e junho de 2024. Amostra aleatória sistematizada de covid-19 grave e leve no período de março de 2020 a janeiro de 2022. A gravidade da covid-19 foi classificada conforme *guideline* norte-americano do Centro de Controle e Prevenção de Doenças Infecciosas. Avaliadas prevalências e realizado teste de Poisson para associação de menor desempenho laboral e dificuldade no retorno ao trabalho e os fatores sociodemográficos, clínicos, assistenciais e funcionais considerados significativos p ≤ 0,05. Dos participantes, 43,9% tiveram menor desempenho e 22,2% tiveram dificuldade no retorno ao trabalho. A covid longa foi classificada por ter dois sintomas ou mais que se desenvolvem e persistem por mais de quatro semanas após covid-19, e não podem ser justificadas por diagnóstico alternativo e avaliada pela escala de Likert para intensidade de sintomas autopercebidos (0–10). A função dos participantes foi avaliada pelas escalas de atividades básicas (Katz), instrumentais (Lawton), implicações funcionais (Whodas), sarcopenia (SARC-F).

**RESULTADOS:**

O menor desempenho no trabalho associou-se com idade avançada (RP = 2,20; IC95% 1,27–3,80), assim como ser o(a) principal provedor(a) (RP = 1,55; IC95% 1,05–2,29), e apresentar comprometimento funcional moderado (RP = 2,99; IC95% 1,60–5,58) ou grave (RP = 4,88; IC95% 2,59–9,19). Para dificuldade no retorno ao trabalho, a principal associação observada foi o comprometimento funcional grave (RP = 4,33; IC95% 1,58–11,80).

**CONCLUSÃO:**

Destaca-se que a idade avançada e, principalmente, a complicação funcional grave significamente associaram-se ao menor desempenho e a dificuldade no retorno ao trabalho na população estudada com covid longa.

## INTRODUÇÃO

O SARS-CoV-2 é um coronavírus que causa a infecção da covid-19 e pode cursar de forma assintomática ou com sintomas leves em aproximadamente 80% dos casos. No entanto, pode provocar uma síndrome respiratória aguda capaz de evoluir para insuficiência respiratória grave em 5% a 10% dos casos^1^. De maneira geral, fatores associados tanto à doença aguda quanto às medidas de confinamento estão relacionados ao enfraquecimento muscular, o que, de certa forma, reduz a capacidade funcional em graus distintos, com impacto na qualidade de vida, na sobrevida^2^ e no trabalho^3^.

A maioria das pessoas com covid-19 percebe a melhora dos sintomas da infecção após alguns dias ou semanas do seu início. No entanto, entre 10% e 20% dos casos, desenvolve-se a covid longa – identificada quando dois ou mais sintomas persistem por mais de quatro semanas após o início da infecção e não podem ser justificados por um diagnóstico alternativo^4^. Essa recuperação tardia dos sintomas, segundo revisões recentes, pode perdurar por mais de sete meses^2,4,5^.

As pessoas que tiveram covid-19 e necessitaram de hospitalização, principalmente aquelas internadas em unidades de terapia intensiva (UTI), apresentam dificuldades funcionais após a alta, incluindo fraqueza muscular grave e prolongada, fadiga, rigidez articular, miopatia, neuropatia de doenças críticas, disfagia, problemas neuropsicológicos e prejuízo da funcionalidade, incluindo piora da marcha e da mobilidade – condições que dificultam o retorno precoce ao trabalho^2^.

Mesmo aqueles trabalhadores que conseguiram retornar às atividades laborais, apesar de apresentarem a covid longa, enfrentam consequências como redução do bem-estar geral, dificuldade para realizar tarefas anteriormente desempenhadas, perda de concentração e menor desempenho de trabalho^6^.

A literatura indica que condições como sexo feminino, idade avançada, presença de doenças crônicas, dispneia e fadiga são fatores que podem impactar o trabalho, assim como dificultar o retorno imediato após o desenvolvimento da forma grave da covid-19^7^. No entanto, são escassos os estudos que relacionam os impactos funcionais da covid longa com o desempenho no trabalho e o retorno às atividades laborais^3^.

Consequentemente, é necessário estimar a prevalência de menor desempenho no trabalho e da dificuldade de retorno às atividades laborais, bem como associá-las com a covid longa, fatores socioeconômicos, clínicos, assistenciais e funcionais em pessoas que tiveram covid-19.

## MÉTODOS

### Tipo e População de Estudo

Foi realizado um estudo transversal em São Paulo, cidade que apresentou o maior número absoluto de casos de covid-19 no Brasil durante o período da pandemia^11^, com dados de pessoas diagnosticadas com covid-19 registrados pela Secretaria Municipal de Saúde entre março de 2020 e fevereiro de 2022, sob aprovação do parecer nº 5.053.884.

Foram notificados 337.420 com diagnóstico de covid-19, tanto graves quanto leves, no município de São Paulo. Os casos graves foram identificados pela “Ficha de Registro Individual do Ministério da Saúde – Casos de Síndrome Respiratória Aguda Grave Hospitalizado”^12^; e os casos leves pela “Ficha de Investigação de SG Suspeito de Doença pelo Coronavírus 2019 (Covid-19)”^13^.

Foram incluídas pessoas com idade igual ou superior a 18 anos, de ambos os sexos, com diagnóstico de covid-19 confirmado por meio do registro de PCR ou teste sorológico positivo, com IgM (Imunoglobulina M) reagente e IgG (Imunoglobulina G) não reagente, que assinaram, de forma eletrônica, o Termo de Consentimento Livre e Esclarecido. Foram excluídas as pessoas que apresentavam informações inconsistentes que impossibilitavam o contato, bem como aquelas que não responderam ao convite para participação no estudo.

O critério de gravidade adotado pela cidade de São Paulo foi o do *guideline* norte-americano do Centro de Controle e Prevenção de Doenças Infecciosas^14^ e do Ministério da Saúde brasileiro. Os casos foram considerados leves quando apresentaram, ou não, sintomas gripais como coriza, tosse, dor de garganta ou dor de cabeça. Para a forma grave da covid-19, foram considerados aqueles que apresentaram um ou mais dos seguintes sinais: desconforto respiratório (frequência respiratória > 30 irpm), saturação periférica < 93% em ar ambiente, dor abdominal difusa e instabilidade hemodinâmica.

A covid longa oriunda dos casos leves e graves foi classificada de acordo com o Ministério da Saúde^4^, sendo identificada quando dois ou mais sintomas persistem por mais de quatro semanas após o início da infecção e não podem ser justificados por um diagnóstico alternativo^4^.

### Amostra

A partir da população de 337.420 casos notificados (distribuídos entre graves e leves), foi realizada uma seleção aleatória de 25.026 pessoas, com distribuição proporcional da população nas seis regiões de saúde do município, para serem convidadas a participar. Destas, 775 pessoas – sendo 395 com diagnóstico de covid-19 leve e 380 com diagnóstico grave – participaram da pesquisa, completando os questionários eletrônicos.

A coleta foi realizada no período entre abril de 2023 e junho de 2024, em média 2,5 anos após a infecção. Os participantes foram convidados a participar do estudo por meio de contato telefônico. Aqueles que aceitaram participar assinaram o Termo de Consentimento Livre e Esclarecido de forma eletrônica e responderam a um questionário autoaplicável, por meio de um *link* enviado pelos pesquisadores, que continha as informações necessárias para preenchimento do formulário, bem como orientações para contato com os pesquisadores, via telefone, em casos de dúvidas ou dificuldade no preenchimento. A segurança e o armazenamento dos dados foram feitos de acordo com a Lei Geral de Proteção de Dados Pessoais.

Para o cálculo do tamanho da amostra, considerou-se uma prevalência esperada de 30% de incapacidade funcional pós-covid-19^15^, com erro máximo aceitável de 4% e nível de confiança de 95%. Com base nesses parâmetros, estimou-se a necessidade de incluir, no mínimo, 504 pessoas no estudo. A amostra final, composta por 775 pessoas com covid-19 leve e grave que completaram os questionários, resultou em um poder amostral de 99,0% para detectar a prevalência esperada.

### Variáveis

A coleta de dados foi realizada por meio de um questionário autoaplicável, disponibilizado eletronicamente pela plataforma RedCap®, e enviado aos participantes que aceitaram o convite. O questionário foi estruturado para coletar diferentes categorias de informações relevantes para o estudo:

- *Dados sociodemográficos*: incluíram idade, sexo, raça/etnia, estado civil, escolaridade, situação laboral, renda familiar e status de principal provedor(a) da família.
- *Dados clínicos*: abrangeram as comorbidades preexistentes à covid-19, a gravidade da infecção aguda por covid-19 (leve ou grave), o tempo desde o início dos primeiros sintomas até a data da entrevista e a presença de covid longa, definida pela persistência de dois ou mais sintomas por mais de quatro semanas após o início da infecção, não explicados por outro diagnóstico.
- *Sintomas pós-covid*^17^: incluíram dor de cabeça, dor muscular, dor articular, desequilíbrio, tontura/vertigem, falta de atenção, indisposição física, fadiga, dispneia, tosse e perda de memória. A intensidade de cada sintoma autopercebido foi quantificada em uma escala de Likert^18^ de 0 a 10, onde 0 representava sintomas muito leves e 10 sintomas muito intensos.
- *Dados assistenciais*: foram coletadas informações sobre internação hospitalar, admissão em UTI e necessidade de suporte ventilatório invasivo durante a fase aguda da covid-19.
- *Avaliação funcional*: o questionário integrou escalas padronizadas para avaliar a capacidade funcional dos participantes no momento da resposta:
- *Índice de Katz*^19^: utilizado para avaliar a independência nas atividades básicas de vida diária (ABVD), como banhar-se, vestir-se, usar o vaso sanitário, continência e alimentação.
- *Escala de Lawton*^20^: aplicada para medir a independência nas atividades instrumentais de vida diária (AIVD), que incluem tarefas mais complexas como usar o telefone, fazer compras, preparar refeições, arrumar a casa, lavar roupas, usar transportes, controlar medicações e manusear dinheiro.
- Whodas 2.0 (*World Health Organization Disability Assessment Schedule* 2.0) versão breve^21^: questionário abrangente sobre funcionalidade que avalia o impacto da condição de saúde nas atividades diárias em diferentes domínios (compreensão e comunicação, mobilidade, autocuidado, relacionamento com as pessoas, atividades de vida e participação).
- SARC-F^22^: instrumento de rastreio para avaliar o risco aumentado de sarcopenia, baseando-se em cinco itens (força, ajuda para caminhar, subir escadas, levantar de uma cadeira e quedas).

### Avaliação do Desempenho Laboral e Retorno ao Trabalho

Os desfechos primários “menor desempenho laboral” e “dificuldade do retorno ao trabalho”, foram avaliados por meio de perguntas dicotômicas (“sim” ou “não”) de autorrelato, inseridas no questionário, e direcionadas apenas aos participantes que relataram ter trabalho remunerado antes da covid-19 e que não estavam aposentados ou desempregados no momento da pesquisa.

*Menor desempenho laboral:* a pergunta formulada foi: “O seu desempenho no trabalho diminuiu após a infecção pela covid-19 por motivo da sua condição física e/ou por sua condição de saúde mental?” A resposta “sim” indicava a presença de menor desempenho.

*Dificuldade de retorno ao trabalho:* a questão foi: “As sequelas físicas e/ou da sua condição de saúde mental pela infecção da covid-19 impediram o seu retorno ao trabalho?” A resposta “sim” indicava a dificuldade.

*Associação entre escalas de avaliação funcional e desfechos laborais:* as escalas de avaliação funcional (Katz, Lawton, Whodas 2.0, SARC-F) foram utilizadas para fornecer uma medida mais estruturada e padronizada do estado funcional dos indivíduos. Embora os desfechos laborais fossem baseados em autorrelato, a inclusão dessas escalas permitiu:

- Corroborar o impacto funcional, verificando se as dificuldades relatadas no trabalho estavam associadas a comprometimentos objetivos na capacidade de realizar atividades básicas e instrumentais da vida diária, bem como no bem-estar funcional geral;
- Identificar fatores contribuintes, analisando quais aspectos específicos da funcionalidade (por exemplo, mobilidade, autocuidado e risco de sarcopenia) tinham maior associação com a percepção de menor desempenho ou dificuldade de retorno ao trabalho;
- Contextualizar os achados, oferecendo uma visão mais abrangente do perfil funcional dos trabalhadores afetados, indo além da percepção individual e incorporando uma dimensão mais clínica à avaliação do impacto da covid longa no contexto laboral.

### Análise Estatística

Os dados foram analisados no Stata 14. As variáveis categóricas foram apresentadas em frequências absolutas e relativas (%), enquanto as variáveis contínuas foram descritas por meio de média e desvio-padrão. A normalidade da distribuição foi avaliada por meio do teste de Shapiro-Wilk. Foram calculados os percentuais e os intervalos de confiança do menor desempenho laboral e dificuldade no retorno ao trabalho, segundo a gravidade da doença e a presença de covid longa.

Foi realizada análise de variância bidirecional (*two-way* ANOVA) para avaliar a associação entre a intensidade dos sintomas persistentes pós-covid-19 (escala de 0 a 10) e o desempenho laboral (influência ou não no menor desempenho), bem como a gravidade da doença (leve ou grave), incluindo o termo de interação entre os fatores.

Assim como para avaliar a associação entre a intensidade dos sintomas persistentes pós-covid-19 (escala de 0 a 10) e a dificuldade de retorno ao trabalho (sim/não), com a gravidade da doença (leve ou grave), incluindo o termo de interação entre os fatores.

Os pressupostos de normalidade dos resíduos e homogeneidade das variâncias foram considerados. Valores de p < 0,05 foram considerados estatisticamente significativos.

As variáveis dependentes foram “menor desempenho laboral”^16^ e “dificuldade para retorno ao trabalho”^3^. Ambas foram avaliadas por meio de pergunta dicotômica (sim ou não). Para o “menor desempenho laboral”, questionou-se se o desempenho no trabalho havia diminuído após a infecção por covid-19 em decorrência de condição física e/ou mental. Para a “dificuldade no retorno ao trabalho”, investigou-se se as sequelas físicas e/ou mentais da covid-19 impediram o retorno às atividades laborais.

As variáveis independentes consideradas incluíram fatores sociodemográficos (idade, sexo, raça, escolaridade, situação laboral, renda familiar, principal provedor(a)), clínicos (comorbidades preexistentes, gravidade da covid-19, tempo de sintomas, presença de covid longa) e assistenciais (internação, admissão em UTI, necessidade de suporte ventilatório).

Reconhece-se que, em estudos transversais, a distinção entre confundidores e mediadores pode ser complexa, uma vez que algumas variáveis (como a renda) podem atuar em vias causais indiretas. As análises multivariadas foram estruturadas para controlar fatores considerados confundidores diretos, conforme especificado na construção dos modelos, bem como para explorar as associações observadas dentro das limitações inerentes ao delineamento transversal.

Foi realizada regressão de Poisson na base logarítmica com variância robusta. Para a construção dos modelos multivariados finais, foram incluídas variáveis que apresentaram p < 0,20 na análise bivariada, além daquelas consideradas potenciais confundidoras *a priori* com base na literatura e relevância epidemiológica (idade, raça, sexo e escolaridade), independentemente de sua significância inicial. Essa abordagem buscou mitigar as limitações de modelos puramente *stepwise*, priorizando a coerência teórica e o controle de confundimento conhecido.

Os modelos finais foram construídos utilizando dois critérios: Modelo 1 com todas as variáveis funcionais: ABVD (Katz); AIVD (Lawton) e implicações funcionais (Whodas) e o Modelo 2 com apenas implicações funcionais (Whodas).

Para a presente análise, foram considerados apenas os questionários eletrônicos completamente preenchidos para as variáveis de interesse (análise de casos completos), não sendo utilizados métodos de imputação para dados faltantes.

## RESULTADOS

Tomando como base os 337.420 casos, participaram deste estudo 775 pessoas, das quais 395 foram classificadas como casos leves de covid-19 (50,9%) e 380 como casos hospitalizados (49,0%). Dentre estes, 240 (63,1%) evoluíram para a forma grave, com manifestação de desconforto respiratório e dessaturação; 14 (3,8%) apresentaram dor abdominal difusa e sintomas gastrointestinais; e 126 (33,1%) evoluíram para forma crítica, necessitando de internação em UTI devido à piora do quadro de base, insuficiência respiratória, hipotensão e/ou arritmias importantes, má perfusão periférica ou quadro febril persistente por mais de 48 horas e alteração do estado mental ou letargia (Figura).

**Figura:** Fluxograma do estudo.

O tempo médio entre o aparecimento dos primeiros sintomas, conforme registro na notificação, até a participação do estudo foi de 29,5 meses (mediana: 29 meses; intervalo interquartil: 11 meses). A média de idade dos participantes desta pesquisa foi de 49 anos (18–93), sendo que 435 (56,1%) eram do sexo feminino.

Conforme ilustrado na Figura, a amostra final de 775 participantes é composta por indivíduos que aceitaram e completaram os questionários a partir de um convite inicial feito a 25.026 selecionados aleatoriamente. Devido às recusas e perdas de contato, a amostra de respondedores pode ser considerada de conveniência, o que constitui uma limitação discutida adiante. No entanto, o tamanho amostral alcançado foi suficiente para garantir um poder estatístico de 99,0% para os desfechos estudados.

Com relação às questões de trabalho, 295 (72,6%) dos casos leves e 263 (69,2%) dos casos graves relataram possuir trabalho remunerado antes da covid-19. Após a infecção, 281 (71,1%) dos leves e 248 (65,2%) dos graves relataram permanecer no mesmo trabalho. Quanto ao tipo de vínculo, entre os casos leves, 231 (58,4%) possuíam emprego formal e 64 (16,2%) emprego informal; entre os casos graves, 188 (49,4%) possuíam emprego formal e 38 (10,0%) emprego informal.

Entre os casos graves, 329 (75,6%) trabalhadores apresentaram covid longa, enquanto, entre os casos leves, 86 (19,7%) apresentaram a mesma condição. Dentre esses, 121 (27,5%) dos casos graves apresentaram menor desempenho laboral, enquanto 100 (22,7%) dos casos leves relataram essa mesma condição.

As Tabelas 1A e 1B apresentam as associações das características sociodemográficas, clínicas, assistenciais e funcionais com o menor desempenho laboral e dificuldade no retorno ao trabalho, respectivamente.

A prevalência do menor desempenho laboral foi de 43,9% (IC95% 39,7–48,2), sendo de 48,6% (IC95% 42,4–54,8) nos casos graves e de 39,6% (IC95% 33,9–45,5) nos casos leves. A dificuldade no retorno ao trabalho foi observada em 22,2% (IC95% 18,8–25,9) dos indivíduos que tiveram covid-19, sendo que 30,1% (IC95% 24,6–36,1) nos casos graves e 15,0% (IC95% 11,0–19,7) nos casos leves. Em relação ao menor desempenho laboral, os casos graves apresentaram uma razão de prevalência de 1,22 em comparação aos casos leves (IC95% 1,01–1,48). Já para a dificuldade no retorno ao trabalho, os casos graves apresentaram uma razão de prevalência duas vezes maior do que os casos leves (IC95% 1,43–2,79). A covid longa, apresentou razão de prevalência de 1,15 para a queda do desempenho laboral (IC95% 1,04–1,46).

O Gráfico 1 apresenta que o menor desempenho laboral esteve significativamente associado ao aumento da intensidade de todos os sintomas avaliados, incluindo dor articular (F = 31,77; p < 0,001), dor muscular (F = 26,86; p < 0,001), tontura/vertigem (F = 12,09; p = 0,0006), dor de cabeça (F = 10,26; p = 0,0016), dispneia (F = 11,52; p = 0,0008), fadiga (F = 72,15; p < 0,001), déficit de atenção (F = 16,60; p < 0,001), perda de memória (F = 19,74; p < 0,001), indisposição física (F = 41,19; p < 0,001), tosse (F = 10,43; p = 0,0015) e desequilíbrio (F = 5,79; p = 0,017).

**Gráfico 1:** Intensidade dos sintomas persistentes pós-covid-19 segundo desempenho laboral e gravidade da doença.

Nota: *boxplots* da intensidade dos sintomas pós-covid-19 segundo desempenho laboral e gravidade. O eixo vertical representa a intensidade autorreferida de cada sintoma (escala de 0 a 10), enquanto o eixo horizontal indica a presença de impacto no desempenho laboral, categorizado como “sim” (houve influência) ou “não” (não houve influência), estratificado conforme a gravidade da covid-19. *Two-way* ANOVA demonstrou efeito significativo do desempenho laboral (p < 0,05), sem interação com a gravidade.

*p < 0,05.

**p < 0,01.

***p < 0,001.

A gravidade da covid-19 apresentou associação significativa com tontura (F = 10,77; p = 0,0012), dor de cabeça (F = 6,58; p = 0,011), falta de atenção (F = 5,05; p = 0,025), perda de memória (F = 5,22; p = 0,023), dispneia (F = 6,50; p = 0,011), fadiga (F = 8,92; p = 0,003) e indisposição física (F = 8,48; p = 0,0039), mas não com dor articular, dor muscular, tosse ou equilíbrio (p > 0,05).

Não houve interação significativa entre desempenho laboral e gravidade da doença para nenhum dos sintomas avaliados (p > 0,05), indicando que o impacto do desempenho laboral sobre a intensidade dos sintomas ocorre de forma independente da gravidade da fase aguda da infecção.

O Gráfico 2 apresenta que a dificuldade de retorno ao trabalho esteve significativamente associada ao aumento da intensidade de diversos sintomas persistentes, incluindo dor articular (F = 5,94; p = 0,015), dor muscular (F = 5,83; p = 0,016), dispneia (F = 5,79; p = 0,017), fadiga (F = 30,55; p < 0,001), falta de atenção (F = 6,96; p = 0,009), perda de memória (F = 5,37; p = 0,021), tosse (F = 6,84; p = 0,010) e indisposição física (F = 17,65; p < 0,001).

**Gráfico 2:** Intensidade dos sintomas persistentes pós-covid-19 segundo dificuldade de retorno ao trabalho e gravidade da doença.

Nota: *boxplots* da intensidade dos sintomas pós-covid-19 segundo dificuldade de retorno ao trabalho e gravidade da doença. O eixo vertical representa a intensidade autorreferida de cada sintoma (escala numérica de 0 a 10). O eixo horizontal indica a presença de dificuldade de retorno ao trabalho, categorizada como “sim” (com dificuldade) ou “não” (sem dificuldade), com estratificação conforme a gravidade da covid-19. *Two-way* ANOVA demonstrou efeito significativo da dificuldade de retorno ao trabalho (p < 0,05), sem interação com a gravidade.

*p < 0,05.

**p < 0,01.

***p < 0,001.

A gravidade da covid-19 apresentou associação significativa com tontura/vertigem (F = 10,28; p = 0,002), dor de cabeça (F = 9,08; p = 0,003), dispneia (F = 5,62; p = 0,019), fadiga (F = 9,74; p = 0,002), indisposição física (F = 5,51; p = 0,020) e desequilíbrio (F = 3,95; p = 0,049).

Não foi observada interação significativa entre dificuldade de retorno ao trabalho e gravidade da doença para nenhum dos sintomas avaliados (p > 0,05), indicando que o efeito da limitação funcional ocorre de forma independente da gravidade da fase aguda.

A Tabela 2 expõe os modelos de regressão de Poisson multivariada para menor desempenho laboral. Observa-se que, independentemente da gravidade e do tempo decorrido desde a infecção, não houve associação para menor desempenho. No entanto, as variáveis: “ser o(a) principal provedor(a)” (RP = 1,55; IC95% 1,05–2,29), “implicações funcionais moderada” (RP = 2,69; IC95% 1,44–5,01) e “grave” (RP = 4,21; IC95% 2,22–7,95) mostraram-se associadas com o menor desempenho laboral, Modelo 1.

**Tabela 2:** Regressão logística multivariada para menor desempenho laboral após a covid-19.

Variável independente		Menor desempenho laboral			
		Modelo 1		Modelo 2	
		RP (IC95%)	p-valor	RP (IC95%)	p-valor
Idade	18–59 anos	1		1	
	> 60 anos	1,22 (0,82–1,81)	0,317	**2,20 (1,27–3,80)**	**0,036**
Sexo	Masculino	1		1	
	Feminino	0,98 (0,72–1,32)	0,902	0,89 (0,69–1,15)	0,396
Raça	Branca	1		1	
	Preta/Parda	0,96 (0,56–1,63)	0,354	0,96 (0,32–1,97)	0,873
	Outras	0,34 (0,08–1,39)	0,959	0,38 (0,76–9,11)	0,181
Escolaridade	Fundamental	1		1	
	Médio	0,87 (0,54–1,40)	0,574	0,96 (0,22–1,24)	0,877
	Superior	1,07 (0,68–1,67)	0,749	1,14 (0,76–1,70)	0,511
Gravidade da covid-19	Leve	1		1	
	Grave	0,84 (0,61–1,16)	0,303	1,01 (0,78–1,28)	0,960
Tempo de sintomas da covid-19 (meses)	15–29	1		1	
	30–50	1,22 (0,92–1,62)	0,166	1,15 (0,89–1,49)	0,281
Covid longa	Sim	1,75 (0,48–6,40)	0,395	1,71 (0,45–6,48)	0,428
Renda	R$ 500,00–R$ 2.000,00	1		1	
	R$ 2.000,00–R$5.000,00	1,11 (0,72–1,71)	0,624	1,30 (074–1,59)	0,662
	R$ 5.000,00–R$ 10.000,00	1,06 (0,64–1,75)	0,794	0,97 (0,60–1,61)	0,850
	> R$ 10.000,00	0,94 (0,55–1,59)	0,829	1,20 (0,58–1,49)	0,780
Principal provedor(a)		1,55 (1,05–2,29**)**	**0,024**	1,30 (1,02–1,66)	**0,028**
Comorbidade preexistente	Hipertensão arterial	1,31 (0,96–1,79)	0,081	1,30 (0,97–1,75)	0,075
	DPOC	1,06 (0,71–1,59)	0,759	0,97 (0,68–1,37)	0,287
	Diabetes	1,15 (0,83–1,59)	0,388	1,20 (0,90–1,60)	0,865
Sarcopenia	Sarcopênico	1,06 (0,83–1,36)	0,597		
ABVD (Katz)	Independente	1			
	Dependente parcial	1,05 (0,73–1,51)	0,761		
	Dependente total	1,14 (0,78–1,67)	0,487		
AIVD (Lawton)	Dependente	1,10 (0,52–2,33)	0,789		
Comprometimento funcional (Whodas)	Leve	1,46 (0,74–2,90)	0,260	1,90 (0,99–3,67)	0,053
	Moderado	2,69 (1,44–5,01)	**0,002**	2,99 (1,60–5,58)	**0,001**
	Grave	4,21 (2,22–7,95)	**< 0,001**	4,88 (2,59–9,19)	**< 0,001**

RP: razão de prevalência; IC95%: intervalo de confiança de 95%; DPOC: doença pulmonar obstrutiva crônica; ABVD: atividades básicas de vida diária logo após a covid-19; AIVD: atividades instrumentais de vida diária logo após a covid-19; Whodas: World Health Organization Disability Assessment Schedule.

Nota: foram construídos o Modelo 1 com todas as variáveis funcionais: ABVD (Katz); AIVD (Lawton) e implicações funcionais (Whodas) e o Modelo 2 com apenas implicações funcionais (Whodas) para melhorar a interpretação e compreensão os fatores que influenciam nos desfechos. Os p-valores em negrito indicam os fatores significativamente associados ao desfecho de menor desempenho laboral (p < 0,05).

No Modelo 2, que inclui apenas domínios do Whodas como variável funcional, a idade avançada (RP = 2,20; IC95% 1,27–3,80) e os comprometimentos moderado e grave da funcionalidade apresentaram maior destaque na associação com o menor desempenho laboral: (RP = 2,99; IC95% 1,60–5,58); p = 0,001 e (RP = 4,88; IC95% 2,59–9,19; p = < 0,001), respectivamente.

A Tabela 3 apresenta dois modelos de regressão de Poisson multivariada para os fatores associados às sequelas que dificultaram o retorno ao trabalho. A principal associação observada foi com o comprometimento funcional grave (RP = 3,91; IC95% 1,72–8,89; p = 0,001). Além disso, fatores como idade avançada e a sarcopenia também estiveram associados à dificuldade no retorno ao trabalho.

**Tabela 3:** Regressão logística multivariada de dificuldade no retorno ao trabalho após a covid-19.

Variável independente		Dificuldade de retorno ao trabalho			
		Modelo 1		Modelo 2	
		RP (IC95%)	p-valor	RP (IC95%)	p-valor
Idade	18–59 anos	1		1	
	> 60 anos	2,18 (1,05–4,49)	0,034	2,20 (1,27–3,80)	**0,036**
Sexo	Masculino	1		1	
	Feminino	1,22 (0,62–2,39)	0,548	1,05 (0,65–1,78)	0,815
Raça	Branca	1		1	
	Preta/parda	0,38 (0,92–2,34)	0,354	0,80 (0,32–1,97)	0,633
	Outras	1,24 (0,68–12,7)	0,959	0,83 (0,76–9,11)	0,883
Escolaridade	Fundamental	1		1	
	Médio	0,66 (0,17–2,43)	0,533	0,52 (0,22–1,24)	0,143
	Superior	1,07 (0,36–3,16)	0,895	0,78 (0,40–1,53)	0,486
Gravidade da covid-19	Leve	1		1	
	Grave	1,68 (0,76–3,74)	0,197	1,49 (0,81–2,73)	0,193
Tempo de sintomas da covid-19 (meses)	15–29	1		1	
	30–50	1,07 (0,56–2,04)	0,829	1,17 (0,63–2,07)	0,527
Covid longa		0,53 (0,12–2,26)	0,393	0,77 (0,19–3,13)	0,724
Renda	R$ 500,00–R$ 2.000,00	1		1	
	R$ 2.000,00–R$ 5.000,00	1,10 (0,48–2,55)	0,806	1,14 (0,58–2,22)	0,690
	R$ 5.000,00–R$ 10.000,00	0,70 (0,23–2,10)	0,533	0,86 (0,37–2,01)	0,731
	> R$ 10.000	0,72 (0,26–1,99)	0,534	0,71 (0,30–1,67)	0,443
Ser principal provedor(a)		1,24 (0,59–2,57)	0,563	1,01 (0,75–1,76)	0,947
Comorbidade preexistente	Hipertensão arterial	1,34 (1,01–3,52)	0,286	1,16 (0,72–1,87)	0,532
	DPOC	0,97 (0,78–2,29)	0,717	1,21 (0,71–2,08)	0,467
	Diabetes	0,83 (0,46–2,02)	0,620	1,35 (0,85–2,14)	0,194
Sarcopenia	Sarcopênico	1,96 (1,08–3,55)	0,026	1,95 (1,18–3,24)	**0,009**
Comprometimento ABVD (Katz)	Independente	1			
	Dependente parcial	1,02 (0,43–2,39)	0,963		
	Dependente total	1,06 (0,57–1,97)	0,854		
AIVD (Lawton)	Dependente	2,36 (0,71–7,79)	0,156		
Comprometimento funcional (Whodas)	Leve	1,24 (0,40–3,83)	0,697	1,34 (0,53–3,37)	0,529
	Moderado	1,97 (0,67–5,78)	0,212	2,15 (0,89–5,20)	0,087
	Grave	4,33 (1,58–11,8)	0,004	3,91 (1,72–8,89)	**0,001**

RP: razão de prevalência; IC95%: intervalo de confiança de 95%; DPOC: doença pulmonar obstrutiva crônica; ABVD: atividades básicas de vida diária logo após a covid-19; AIVD: atividades instrumentais de vida diária logo após a covid-19; Whodas: World Health Organization Disability Assessment Schedule.

Nota: Foi construído o Modelo 1 com todas as variáveis funcionais: ABVD (Katz); AIVD (Lawton) e implicações funcionais (Whodas) e o Modelo 2 com apenas SARC-F e Whodas para melhorar a interpretação e compreensão dos fatores que influenciam nos desfechos. Os p-valores em negrito indicam os fatores associados ao desfecho de dificuldade de retorno ao trabalho (p < 0,05).

## DISCUSSÃO

Este estudo mostra que fatores sociodemográficos, clínicos e funcionais estão associados às alterações nas atividades laborais, tanto no desempenho quanto no retorno do trabalho. Fatores como: idade avançada, ser o(a) principal provedor(a), ter comprometimento funcional moderado ou grave estiveram associados ao menor desempenho laboral. Além disso, idade avançada, sarcopenia e comprometimento funcional grave estiveram associados ao impedimento do retorno ao trabalho.

De acordo com os achados deste estudo, o tempo entre o início dos sintomas e a data de preenchimento da pesquisa não apresentou uma associação significativa no menor desempenho de trabalho nem com o retorno laboral. Porém, quanto maior a idade (> 60 anos), a razão de prevalência chega a 2,20 vezes para essa população. Esses resultados corroboram os encontrados nos estudos de Chen et al.^22^ e Westerlind et al.^9^, nos quais a idade avançada foi um preditor de piores desfechos de saúde e dificuldade ou prolongamento do tempo de retorno ao trabalho. Uma dessas hipóteses para esse fenômeno é a imunossenescência – processo de deterioração gradual do sistema de defesa, decorrente do envelhecimento – que leva a uma maior vulnerabilidade a infecções e a uma resposta menos eficaz às vacinações^23^.

Esperava-se que a gravidade da covid-19 estivesse associada ao menor desempenho laboral e dificuldade no retorno ao trabalho; entretanto, isso não foi observado.

A covid longa é mais comum em adultos do sexo feminino, com diabetes mellitus tipo 2, idade avançada, presença de comorbidades preexistentes, esquema vacinal incompleto para covid-19, baixa renda, menor escolaridade, maior gravidade da fase aguda, com internação em UTI^24^. Azevedo et al.^3^, por sua vez, mostraram, em seu estudo de coorte que, embora a maioria dos pacientes tenha retornado ao trabalho após a alta, aproximadamente 70% apresentavam sintomas relacionados à covid longa entre seis e nove meses após a infecção. Nossos achados transversais, embora não permitam a mesma inferência longitudinal, são consistentes com a persistência de sintomas observados na literatura.

Este estudo identificou prevalência de 75,6% de trabalhadores com covid longa nos casos graves e 19,7% entre aqueles com casos leves. Neste estudo, a covid longa, independentemente da gravidade e do tempo de sintomas, não apresentou uma associação significativa com o menor desempenho laboral e nem para dificuldade no retorno ao trabalho. No entanto, os resultados transversais indicam que, no momento da coleta de dados, muitos trabalhadores que relataram covid longa também enfrentavam dificuldades no desempenho laboral ou no retorno ao trabalho, o que evidencia a complexidade das interações entre a condição de saúde e o ambiente de trabalho.

É importante reconhecer que essa prevalência pode ter sido influenciada por viés de seleção, uma vez que indivíduos com sintomas persistentes (covid longa) podem ter tido maior propensão a participar do estudo. Consequentemente, a interpretação dessa variável deve ser feita com cautela, compreendendo-a como uma característica da amostra que pode estar associada aos desfechos, sem permitir inferências causais diretas.

Embora a maioria dos participantes apresentasse renda superior a R$ 2.000,00 e alta escolaridade (ensino superior completo), observou-se uma associação significativa entre ser o(a) principal provedor(a) com o menor desempenho laboral. Esse achado corrobora o estudo de Bejot et al.^28^, que destaca a pressão social exercida sobre um único membro responsável pelo sustento familiar. Essa responsabilidade financeira concentrada pode prejudicar o desempenho no trabalho e contribuir para uma desvantagem cumulativa, favorecendo dificuldades financeiras e menor acesso a cuidados de saúde no longo prazo^29^.

Quanto às sequelas da covid-19, intervenções precoces têm sido associadas à melhora da recuperação e da qualidade de vida^30^. A reabilitação domiciliar ou ambulatorial pode ser necessária para o tratamento das sequelas, e a melhora de sintomas persistentes, como fadiga, pode influenciar diretamente no retorno mais rápido às atividades laborais^31^.

O estudo de Van Wambeke et al*.*^32^ identificou que comorbidades como hipertensão arterial sistêmica, diabetes mellitus e doença pulmonar obstrutiva crônica desempenham um papel significativo na piora funcional dos pacientes, afetando diretamente o desempenho do trabalho e o tempo necessário para o retorno às atividades laborais após a covid-19, o que difere deste estudo, no qual as comorbidades preexistentes (hipertensão arterial, doença pulmonar obstrutiva crônica e diabetes) não se associaram com o menor desempenho e nem para a dificuldade no retorno ao trabalho.

Em relação às possíveis consequências pós-infecção, Morley^33^ descreve que sobreviventes da covid-19 podem apresentar piora da função muscular, com aumento do risco de sarcopenia aguda. Em pacientes em estado grave, internados e sob ventilação mecânica, a redução do trofismo muscular pode atingir até 2% por dia^34^.

Outros fatores que contribuem para a sarcopenia são o isolamento social e o aumento do trabalho remoto, adotados como medidas de contenção da disseminação do vírus^34^. O estudo de Silva et al.^35^ demonstrou aumento de 26,0% na inatividade física durante esse período. No presente estudo, a sarcopenia esteve presente em 22,7% com menor desempenho laboral e 26,6% que tiveram dificuldade no retorno ao trabalho, estando associada a um aumento de até 1,96 vez para dificultar o retorno ao trabalho.

Além das sequelas pós-covid, destacou-se a associação entre o comprometimento funcional moderado a grave para menor desempenho do trabalho e o comprometimento funcional grave associado à dificuldade no retorno ao trabalho. Vanichkachorn et al.^36^, em uma coorte, descrevem a covid longa como uma condição que pode persistir por meses após a infecção inicial, resultando em comprometimento funcional significativo. Esses fatores clínicos e funcionais podem impactar negativamente a capacidade funcional e a qualidade de vida, além de dificultar o retorno ao trabalho. Com isso, os resultados presentes complementam essa visão ao levantar a hipótese da associação entre limitações funcionais e menor desempenho laboral e dificuldade no retorno ao trabalho.

Portanto, este estudo destaca a necessidade de ampliar o olhar sobre os indivíduos acometidos pelas sequelas da covid-19, considerando não apenas os aspectos físicos, mas também os fatores sociais, econômicos e funcionais associados ao menor desempenho e dificuldade no retorno ao trabalho. Independentemente da gravidade e do tempo de sintomas, foram observadas associações com prejuízos nas atividades laborais. Intervenções oportunas, por meio de ações intersetoriais em saúde, trabalho e reabilitação interdisciplinar, podem favorecer tanto o retorno quanto a melhora do desempenho laboral.

Do ponto de vista das limitações, este trabalho foi realizado de maneira autoaplicada entre 15 e 50 meses após a infecção inicial por covid-19. Desse modo, pode ter ocorrido viés de memória. Reconhecemos que ocorreu um possível viés de seleção em face da alta taxa de não respondentes com a insegurança dos participantes em responderem à pesquisa por telefone. Uma limitação adicional é que, devido à natureza autoaplicada do questionário e à impossibilidade de comparar características sociodemográficas ou clínicas entre os participantes e os não respondedores a partir dos dados secundários disponíveis, não foi possível investigar potenciais vieses de seleção relacionados a essas características básicas (idade, sexo, escolaridade) que poderiam ter influenciado os resultados. Esta falta de informação pode limitar a generalização dos nossos achados.

Diante das incertezas relacionadas aos impactos a longo prazo da pandemia de covid-19, recomenda-se a realização de estudos longitudinais para investigar os efeitos sociais, clínicos, assistenciais e funcionais, bem como sua relação com o desempenho e o retorno ao trabalho, permitindo o estabelecimento de relações causais mais robustas.

## CONCLUSÃO

As pessoas que tiveram covid-19 nas formas grave e leve retornaram às atividades laborais, no entanto, quase metade apresentou menor desempenho laboral. A proporção do prejuízo do desempenho no trabalho foi maior entre os casos graves, e a covid longa não teve associação com o menor desempenho do trabalho nem para a dificuldade no retorno ao trabalho.

Fatores como idade avançada e comprometimento funcional moderado a grave estiveram associados ao menor desempenho das atividades laborais. Por outro lado, a dificuldade no retorno ao trabalho foi associada principalmente à idade avançada, sarcopenia e ao comprometimento funcional grave.

Dessa forma, destaca-se a importância de ações intersetoriais entre saúde e trabalho, com ênfase nas necessidades assistenciais individuais e coletivas desses trabalhadores para otimizar a capacidade funcional e melhorar a qualidade de vida no trabalho.

**Tabela 1:** Características sociodemográficas, clínicas, assistenciais e funcionais para o desempenho laboral e no retorno ao trabalho.

Característica	Menor desempenho laboral			Sem influência no desempenho laboral			p-valor^a^
	Grave	Leve	Total	Grave	Leve	Total	
	n (%)	n (%)		n (%)	n (%)		
**Sociodemográfica**							
Idade em anos							0,659
18–59	69 (47,5)	95 (95,9)	164 (79,6)	76 (68,5)	132 (91,0)	208 (81,2)	
> 60	38 (52,5)	4 (4,1)	42 (20,4)	35 (31,5)	13 (9,0)	48 (18,8)	
Sexo							0,405
Masculino	70 (56,0)	36 (31,8)	106 (43,9)	86 (63,7)	60 (34,9)	146 (47,5)	
Feminino	58 (44,0)	77 (68,2)	135 (56,1)	49 (36,3)	112 (65,1)	161 (52,5)	
Raça							0,888
Branca	84 (66,7)	55 (49,5)	139 (58,6)	94 (70,1)	90 (53,2)	184 (60,7)	
Preta/Parda	15 (11,9)	19 (17,1)	34 (14,3)	11 (8,2)	31 (18,3)	42 (13,8)	
Outras	27 (21,4)	37 (33,4)	64 (27,1)	29 (21,7)	48 (28,5)	77 (25,5)	
Estado Civil							0,757
Solteiro	19 (15,4)	45 (39,8)	64 (27,0)	26 (19,5)	66 (59,5)	92 (30,7)	
Casado	85 (68,5)	53 (46,9)	138 (58,2)	81 (61,0)	80 (60,2)	161 (53,7)	
Divorciado	14 (11,3)	14 (12,4)	28 (11,8)	19 (14,3)	19 (57,6)	38 (12,7)	
Viúvo	6 (4,8)	1 (0,9)	7 (3,0)	7 (5,2)	2 (66,6)	9 (2,9)	
Escolaridade							0,551
Fundamental	12 (17,1)	9 (11,5)	21 (14,1)	9 (9,9)	8 (6,7)	17 (8,1)	
Médio	12 (17,1)	28 (34,6)	40 (27,0)	12 (13,2)	44 (37,0)	56 (27,0)	
Superior	46 (65,8)	41 (52,5)	87 (58,7)	70 (76,9)	67 (56,3)	137 (64,9)	
Situação laboral							0,251
Formal	73 (60,8)	74 (66,0)	147 (63,3)	96 (72,7)	121 (68,7)	217 (71,6)	
Informal	16 (13,3)	15 (13,4)	31 (12,8)	7 (5,3)	21 (11,9)	28 (9,2)	
Renda							**0,006**^a^
Até R$ 2.000,00	12 (11,2)	40 (37,4)	52 (24,3)	12 (10,2)	32 (20,4)	44 (16,0)	
R$ 2.000,000–R$ 5.000,00	42 (39,2)	44 (41,1)	86 (40,2)	26 (22,0)	66 (42,0)	92 (33,4)	
R$ 5.000,000–R$ 10.000,00	22 (20,6)	13 (12,1)	35 (16,3)	35 (29,7)	32 (20,4)	67 (24,4)	
> R$ 10.000,00	31 (29,0)	10 (9,4)	41 (19,2)	45 (38,1)	27 (17,2)	72 (26,2)	
Principal provedor(a)	99 (34,6)	86 (30,0)	185 (64,6)	36 (12,5)	65 (22,9)	101 (35,4)	**0,013**^a^
**Clínica**							
Comorbidade							
Hipertensão arterial	42 (26,4)	25 (28,0)	67 (27,0)	30 (24,0)	25 (25,5)	55 (24,7)	**0,006**^a^
Diabetes	27 (17,0)	9 (10,1)	36 (14,5)	21 (16,8)	10 (10,2)	31 (13,9)	**0,006**^a^
Obesidade	67 (42,1)	38 (42,7)	105 (42,3)	59 (47,2)	55 (56,1)	114 (51,1)	0,132
DPOC	23 (14,5)	17 (19,2)	40 (16,2)	15 (12,0)	8 (8,2)	23 (10,3)	**0,001**^a^
Tempo de sintomas (meses)							0,515
15–29	52 (21,5)	70 (29,0)	122 (50,5)	49 (16,4)	115 (36,0)	156 (52,4)	
30–50	76 (31,5)	43 (18,0)	119 (49,5)	86 (28,8)	57 (18,8)	142 (47,6)	
Covid longa	121 (27,5)	100 (22,7)	221 (50,2)	105 (23,8)	114 (26,0)	219 (49,8)	**0,012**^a^
Assistencial							
Hospitalizado	127 (73,8)	-	127 (73,8)	135 (76,3)	-	135 (76,3)	0,304
UTI	45 (26,4)	-	45 (26,4)	42 (23,7)	-	42 (23,7)	0,414
Suporte ventilatório					-	-	0,076
Invasivo	12 (15,6)	-	12 (15,6)	7 (9,2)	-	7 (9,2)	
Não invasivo	65 (84,4)	-	65 (84,4)	69 (90,8)	-	69 (90,8)	
**Funcional**							
Atividade básica de vida diária (logo após a covid-19)							**< 0,001**^a^
Função completa	50 (52,1)	90 (83,3)	140 (68,6)	95 (76,0)	144 (92,4)	239 (85,0)	
Dependentes parcial	14 (14,6)	12 (11,1)	26 (12,7)	17 (13,6)	6 (3,8)	23 (8,2)	
Dependente total	32 (33,3)	6 (5,6)	38 (18,7)	13 (10,4)	6 (3,8)	19 (6,8)	
Atividade instrumental de vida diária (logo após a covid-19)							**0,010**^a^
Independente	111 (81,7)	102 (98,0)	213 (88,7)	167 (93,3)	123 (96,8)	290 (94,8)	
Dependente	25 (18,3)	2 (2,0)	27 (11,3)	12 (6,7)	4 (3,2)	16 (5,2)	
Sarcopenia							**< 0,001**^a^
Eutrófico	90 (78,2)	77 (76,2)	167 (77,3)	124 (96,1)	138 (95,2)	262 (95,6)	
Sarcopênico	25 (21,8)	24 (23,8)	49 (22,7)	5 (3,9)	7 (4,8)	12 (4,4)	
Whodas comprometimento funcional							**< 0,001**^a^
Leve	9 (7,6)	8 (7,7)	17 (7,6)	66 (50,8)	53 (37,1)	119 (42,0)	
Moderado	63 (52,9)	49 (47,1)	112 (50,2)	55 (42,3)	89 (62,2)	144 (50,8)	
Grave	47 (39,5)	47 (45,2)	94 (42,2)	9 (6,9)	11 (0,7)	20 (7,2)	
**Característica**	**Dificuldade no retorno ao trabalho**			**Sem dificuldade de retorno ao trabalho**			**p-valor^a^**
	**Grave**	**Leve**	**Total**	**Grave**	**Leve**	**Total**	
	**n (%)**	**n (%)**		**n (%)**	**n (%)**		
**Sociodemográfica**							
Idade em anos							**0,002**^a^
18–59	35 (55,5)	33 (94,3)	68 (69,4)	109 (70,8)	195 (92,8)	314 (83,9)	
> 60	28 (44,5)	2 (5,7)	30 (30,6)	45 (29,2)	15 (7,2)	60 (16,1)	
Sexo							0,523
Masculino	42 (53,2)	11 (25,6)	53 (43,5)	111 (91,0)	85 (35,0)	196 (53,7)	
Feminino	37 (46,8)	32 (74,4)	69 (56,5)	11 (9,0)	158 (65,0)	169 (46,3)	
Raça							0,626
Branca	49 (83,0)	19 (79,2)	68 (81,9)	128 (88,9)	125 (73,1)	253 (80,6)	
Preta/Parda	10 (17,0)	5 (20,8)	15 (18,1)	16 (11,1)	45 (26,3)	61 (19,4)	
Outras	-	-	-	-	1 (0,6)	-	
Estado Civil							0,809
Solteiro	13 (17,1)	20 (46,5)	33 (27,7)	33 (18,3)	93 (39,1)	126 (30,1)	
Casados	48 (63,1)	19 (44,2)	67 (56,3)	117 (65,0)	114 (47,9)	231 (55,3)	
Divorciado	11 (14,5)	3 (6,9)	14 (11,7)	21 (11,6)	29 (12,2)	50 (12,0)	
Viúvo	4 (5,3)	1 (2,4)	5 (4,3)	9 (5,1)	2 (0,8)	11 (2,6)	
Escolaridade							**0,013**^a^
Fundamental	12 (25,0)	3 (10,3)	15 (19,5)	9 (7,4)	14 (8,3)	23 (7,9)	
Médio	8 (16,6)	12 (41,4)	20 (26,0)	25 (20,7)	61 (36,1)	86 (29,6)	
Superior	28 (58,4)	14 (48,3)	42 (54,5)	87 (71,9)	94 (55,6)	181 (62,5)	
Situação laboral							**< 0,001**^a^
Formal	36 (48,6)	22 (52,4)	58 (50,0)	133 (75,1)	175 (72,3)	312 (73,7)	
Informal	10 (13,5)	6 (14,3)	16 (13,8)	13 (7,3)	30 (12,4)	43 (10,2)	
Renda							**< 0,001**^a^
Até R$2.000,00	11 (17,7)	18 (45,0)	29 (20,6)	12 (7,4)	55 (24,5)	67 (17,8)	
R$ 2.000,000–R$5.000,00	26 (41,9)	16 (40,0)	42 (45,6)	43 (26,4)	94 (42,0)	137 (36,3)	
R$ 5.000,000–R$10.000,00	12 (19,3)	4 (10,0)	16 (17,4)	45 (27,6)	40 (17,8)	85 (22,5)	
> R$ 10.000,00	13 (21,1)	2 (5,0)	15 (16,4)	63 (38,6)	35 (15,7)	88 (23,4)	
Principal provedor(a)	56 (26,4)	33 (15,5)	89 (41,9)	42 (19,8)	81 (38,3)	123 (58,1)	0,718
**Clínica**							
Comorbidade							
Hipertensão arterial	24 (24,7)	38 (56,7)	62 (37,8)	12 (7,9)	48 (30,4)	60 (19,3)	**0,029**^a^
Diabetes	19 (19,6)	6 (8,9)	25 (15,2)	30 (19,7)	14 (8,8)	44 (14,2)	**0,003**^a^
Obesidade	40 (41,2)	15 (22,4)	55 (33,5)	86 (56,6)	79 (50,0)	165 (53,2)	0,167
DPOC	14 (14,4)	8 (12,0)	22 (13,5)	24 (15,8)	17 (10,8)	41 (13,3)	**0,010**^a^
Tempo de sintomas (meses)							0,892
15–29	38 (31,1)	27 (22,1)	65 (53,2)	63 (27,8)	161 (25,2)	224 (53,0)	
30–50	41 (33,6)	16 (13,2)	57 (46,8)	120 (28,0)	82 (19,0)	202 (47,0)	
Covid longa	75 (17,0)	36 (8,2)	111 (25,2)	150 (34,1)	179 (30,7)	329 (74,8)	0,133
Assistencial							
Hospitalizado	79 (71,8)	-	79 (71,8)	182 (61,3)	-	182 (61,3)	0,510
UTI	31 (28,2)	-	31 (28,2)	115 (38,7)	-	115 (38,7)	0,250
Suporte ventilatório							**0,001**^a^
Invasivo	13 (25,5)	-	13 (25,5)	6 (5,9)	-	6 (5,9)	
Não invasivo	38 (74,5)	-	38 (74,5)	96 (94,1)	-	96 (94,1)	
**Funcional**							
Atividade básica de vida diária (logo após a covid-19)							**< 0,001**^a^
Função completa	25 (47,2)	30 (75,0)	55 (61,1)	119 (71,2)	203 (91,0)	322 (82,3)	
Dependente parcial	4 (7,5)	6 (15,0)	10 (11,1)	27 (16,2)	13 (5,8)	40 (10,2)	
Dependente total	24 (45,3)	4 (10,0)	28 (27,8)	21 (12,6)	8 (3,2)	29 (7,5)	
Atividade instrumental de vida diária (logo após a covid-19)							**< 0,001**^a^
Independente	52 (69,3)	36 (92,3)	88 (77,2)	165 (92,2)	221 (98,7)	386 (95,8)	
Dependente	23 (30,7)	3 (6,7)	26 (22,8)	14 (7,8)	3 (1,3)	17 (4,2)	
Sarcopenia							**< 0,001**^a^
Eutrófico	55 (78,5)	25 (64,1)	80 (73,4)	158 (91,3)	190 (91,8)	248 (88,6)	
Sarcopênico	15 (21,5)	14 (35,9)	29 (26,6)	15 (8,7)	17 (8,2)	32 (11,4)	
Whodas comprometimento funcional							**< 0,001**^a^
Leve	7 (9,6)	2 (5,0)	9 (8,0)	67 (38,3)	58 (26,7)	125 (31,9)	
Moderado	37 (50,7)	13 (32,5)	50 (44,2)	80 (45,7)	126 (58,1)	206 (52,5)	
Grave	29 (39,5)	25 (62,5)	54 (47,8)	28 (16,0)	33 (15,2)	61 (15,6)	

UTI: unidade de terapia intensiva; Whodas: *World Health Organization Disability Assessment Schedule*; DPOC: doença pulmonar obstrutiva crônica.

Nota: Os valores com associação estatisticamente significativa estão destacados em negrito.

^a^ Associação estatisticamente significativa (p < 0,05) entre fatores sociodemográficos, clínicos, assistenciais e funcionais e o menor desempenho laboral, bem como o impedimento do retorno ao trabalho, em comparação ao grupo sem influência sobre o desempenho laboral e sem impedimento para o retorno ao trabalho (teste do qui-quadrado).
